# Large-scale metabarcoding analysis of epipelagic and mesopelagic copepods in the Pacific

**DOI:** 10.1371/journal.pone.0233189

**Published:** 2020-05-14

**Authors:** Junya Hirai, Aiko Tachibana, Atsushi Tsuda

**Affiliations:** 1 Atmosphere and Ocean Research Institute, The University of Tokyo, Chiba, Japan; 2 Department of Ocean Sciences, Tokyo University of Marine Science and Technology, Tokyo, Japan; IRIG-CEA Grenoble, FRANCE

## Abstract

A clear insight into the large-scale community structure of planktonic copepods is critical to understanding the mechanisms controlling diversity and biogeography of marine taxa in terms of their high abundance, ubiquity, and sensitivity to environmental changes. Here, we applied a 28S metabarcoding approach to large-scale communities of epipelagic and mesopelagic copepods at 70 stations across the Pacific Ocean and three stations in the Arctic Ocean. Major patterns of community structure and diversity, influenced by water mass structures, agreed with results from previous morphology-based studies. However, a large-scale metabarcoding approach could detect community changes even under stable environmental conditions, including changes in the north/south subtropical gyres and east/west areas within each subtropical gyre. There were strong effects of the epipelagic environment on mesopelagic communities, and community subdivisions were observed in the environmentally stable mesopelagic layer. In each sampling station, higher operational taxonomic unit (OTU) numbers and lower phylogenetic diversity were observed in the mesopelagic layer than in the epipelagic layer, indicating a recent rapid increase in species numbers in the mesopelagic layer. The phylogenetic analysis utilizing representative sequences of OTUs revealed trends of recent emergence of cold-water OTUs, which are mainly distributed at high latitudes with low water temperatures. Conversely, the high diversity of copepods at low latitudes was suggested to have been formed through long evolution under high water temperature conditions. The metabarcoding results suggest that evolutionary processes have strong impacts on current patterns of copepod diversity, and support the “out of the tropics” theory explaining latitudinal diversity gradients of copepods. Diversity patterns in both epipelagic and mesopelagic copepods was highly correlated to sea surface temperature; thus, predicted global warming may have a significant impact on copepod diversity in both layers.

## Introduction

Zooplankton play a significant role as secondary or tertiary producers in pelagic communities, and their diversity is important in supporting the functions of marine ecosystems [1]. Approximately 7,000 species of marine zooplankton species in 15 phyla have been described, and many undocumented zooplankton have been recorded with molecular techniques [2, 3]. Marine planktonic copepods are an important class of zooplankton with approximately 2,700 species of diversity [4]. Their high abundance, ubiquitous distribution, and sensitivity to environmental changes also make copepods good indicators in marine ecosystems [5]. Marine ecosystems are altered rapidly under climate change [6], and modifications in the biogeography of key copepod species have already affected higher-trophic levels, including commercially important fish [7]. Insights into the mechanisms that maintain copepod community structure and diversity would thus contribute to our understanding of broad-scale diversity patterns and monitoring changes in ocean ecosystems.

The Pacific Ocean, which covers more than 30% of the Earth’s surface, is the largest ocean basin, providing numerous habitats for different marine organisms in complex ecosystems [8, 9]. The Pacific Ocean was formed by Panthalassa about 160 million years ago (Ma), making it the oldest ocean basin [10] with a long evolutionary history of marine taxa. In coastal marine ecosystems, for example, the western side of the Pacific facing the Coral Triangle is known as a hotspot of coastal marine species diversity [11]. A high diversity of oceanic copepods is thus expected in the Pacific; however, large-scale studies of copepod communities have mainly been restricted to the Atlantic Ocean [12–14]. The high diversity of copepods in low latitudes with high water temperatures and low productivity has been empirically estimated at a global scale, but those estimations are mainly based on Atlantic data [15]. Early Pacific studies surveyed only the distribution of zooplankton, including copepods, and major water mass structures were a key factor in determining zooplankton biogeography [16, 17]. The whole copepod community was analyzed only in a specific region, and a relatively few large-scale studies were conducted in the Pacific, such as those that compared the copepod community between north and south subtropical circulation [18], or studied the latitudinal patterns of copepods from the equator to subarctic at 160°E [19]. Calanoid community and its diversity were investigated from epipelagic (0–200 m) to bathypelagic (1,000–4,000 m) layers at 12 stations in the North Pacific [20], whereas large-scale studies on copepod communities across the Pacific are limited below the epipelagic layer. Due to climate changes, the pelagic ecosystem is experiencing rapid changes [21]. A high-resolution large-scale analysis of the whole copepod community in the Pacific Ocean and the adjacent Arctic Ocean with high horizontal and vertical coverage will help to elucidate those changes.

Large-scale studies of the copepod diversity have been limited because of difficulties in collecting samples over large areas. Previous large-scale studies have required a high degree of expertise in morphological classifications, as copepod classification at the species level remains incomplete. Molecular techniques with high taxonomic resolution are an effective tool for analyzing copepod diversity in the epipelagic and mesopelagic layers, revealing cryptic and undescribed species in the open ocean [22, 23]. In particular, metabarcoding, which uses a large number of sequence data generated by high-throughput sequencers, is a promising technique to comprehensively identify zooplankton communities [24, 25]. This technique has already been applied to copepods using the Roche 454 high-throughput sequencer and revealed a broad-scale diversity and biogeography of copepods in the epipelagic layer of tropical and subtropical regions in the Pacific based on 28S rRNA gene sequences [26, 27]. The metabarcoding method of copepods was further developed for use with the Illumina MiSeq platform, leading to low-cost and high-quality analyses of the marine planktonic copepod diversity with high taxonomic resolutions [28]. In addition, metabarcoding analysis provides nucleotide sequences of operational taxonomic units (OTUs), which are expected to reveal large-scale evolutionary processes and the underlying patterns of copepod diversity. The global patterns of community structure and diversity have been analyzed using metabarcoding analysis of the 18S rRNA gene in eukaryotic organisms including copepods [3, 29]. Nevertheless, the metabarcoding approach is not yet to be implemented for large-scale Pacific regions.

**Fig 1:**
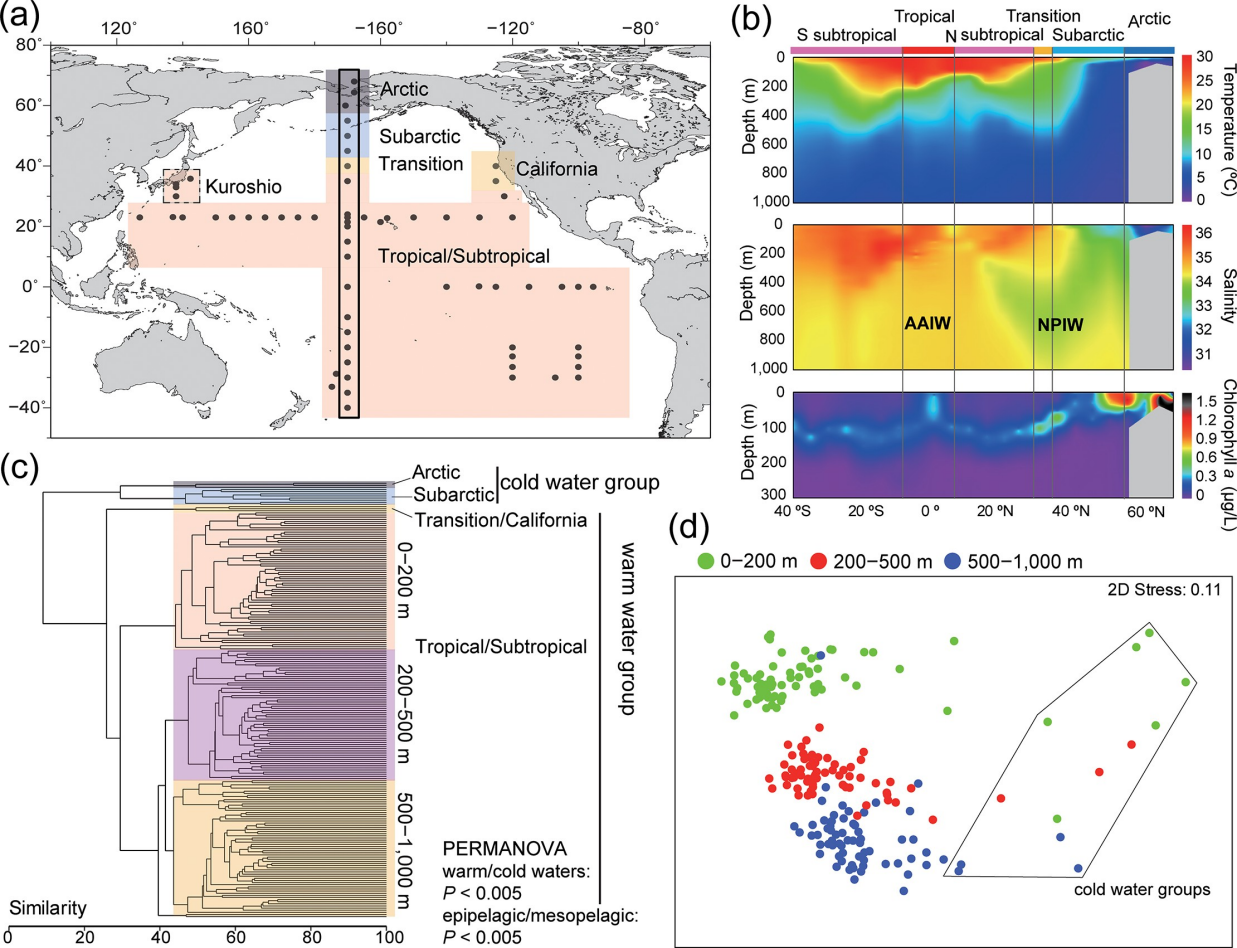
Community composition of copepods based on the presence/absence of operational taxonomic units (OTUs). (a) Sampling location. (b) Vertical profiles of environmental variables across latitudes (top: seawater temperature, middle: salinity, and bottom: chlorophyll *a*) obtained during north–south transect cruises along 170°W (points encircled in black lines in [a]); the North Pacific Intermediate Water (NPIW) and Antarctic Intermediate Water (AAIW) are shown in the salinity profile. (c) Cluster analysis of all copepod communities. Color code for warm-, Transition/California-, and cold-water communities in the epipelagic layer is illustrated in (a). (d) Multidimensional scaling analysis of all samples. Cold-water communities are encircled by black line. Details of the PERMANOVA are presented in S3 Table. Map was created by the authors in Generic Mapping Tools version 5 [30].

In this study, we aimed to investigate the large-scale patterns of community structure and diversity of copepods based on genetic data using a metabarcoding approach in the Pacific Ocean and the neighboring Arctic Ocean. From 2011 to 2017, zooplankton samples were collected from the epipelagic layer (0–200 m), and the upper (200–500 m) and lower (500–1,000 m) mesopelagic layers at 70 stations in the Pacific Ocean, as well as from the epipelagic layer at three stations in the Arctic Ocean (Fig 1A; S1 Table). The study area extended from 40°S to 68°N and from 40°E to 68°W and covered Arctic, Subarctic, Transition, California Current, Kuroshio Current, North subtropical, South subtropical, and Tropical regions. We performed metabarcoding using a high-throughput Illumina MiSeq sequencer on 205 zooplankton samples and conducted a broad-scale comparison of copepod communities based on OTU data to reveal both vertical and horizontal patterns of copepod diversity. Subsequently, the environmental variables explaining copepod communities and diversity were evaluated. We also studied sequence diversity and phylogenetic relationships of the OTUs to reveal the effects of evolutionary processes on diversity and biogeography of copepods in the Pacific Ocean.

## Results

### Metabarcoding data

**Fig 2:**
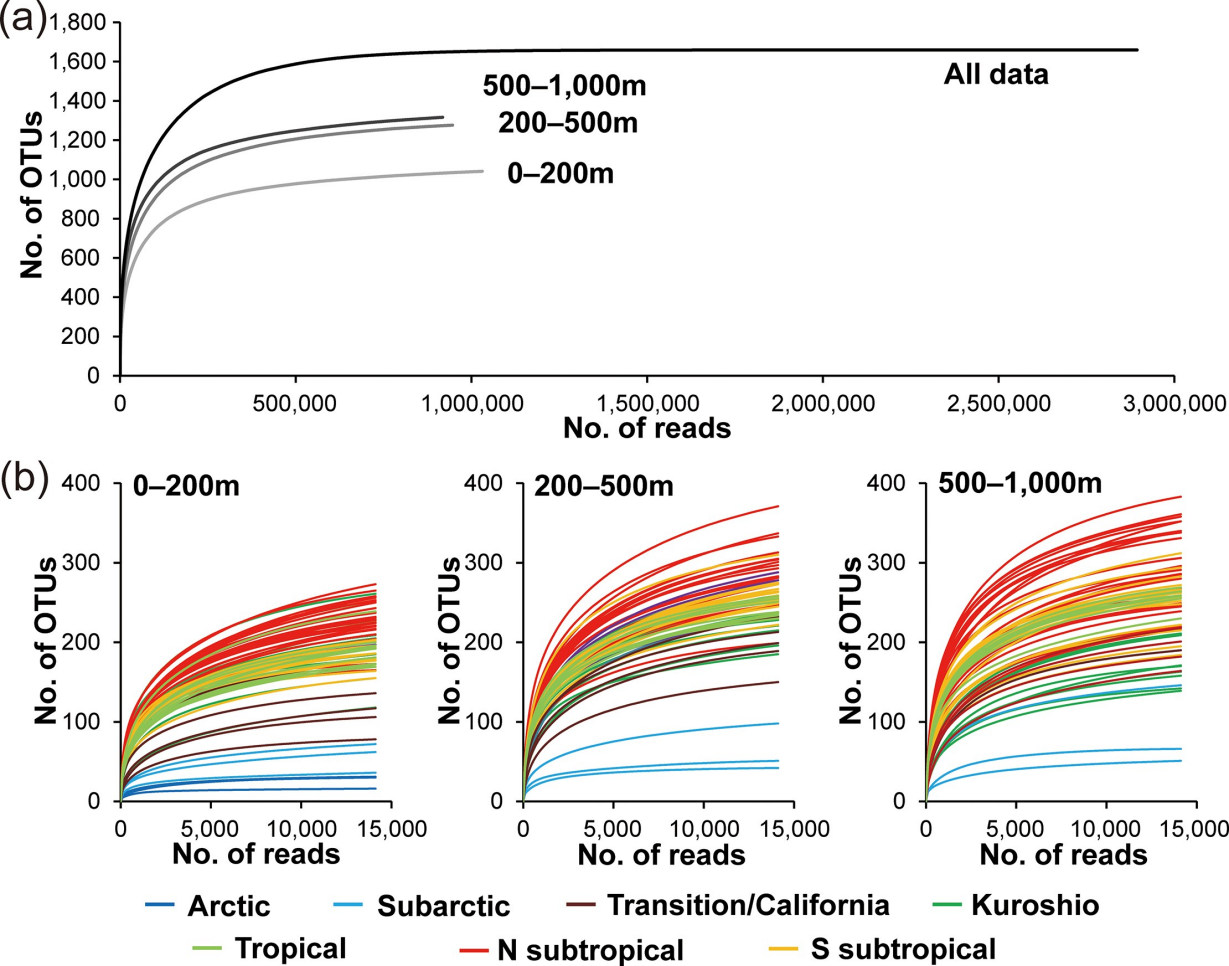
Rarefaction curves for operational taxonomic units (OTUs) at different sequence reads. (a) Results for all sequence reads and total sequence reads at each sampling layer (0–200 m, 200–500 m, and 500–1,000 m). (b) Results for each sample. Geological areas are represented by color.

Sequence reads were clustered into OTUs at 98.5% sequence similarity threshold and a minimum sequence reads of ≥8 to avoid overestimating diversity and maintain optimal taxonomic resolution. These threshold values were determined based on the preliminary analyses using mock communities (S2 Table). The mock communities contained known copepod species, and the analysis compared the OTUs under different similarity and abundance thresholds with the existing copepod reference sequences in GenBank. The mock community data samples were analyzed together with all sequence data of environmental communities to validate the accuracy of data analysis (S1 Fig). A total of 14,117 sequence reads were obtained from each sample after a standardization of environmental community analyses, and 2,893,985 copepod sequence reads were clustered into 1,659 copepod OTUs. The rarefaction curve using all sequence data showed that the numbers of OTUs reached a plateau (Fig 2A). Not all rarefaction curves of each sample at each sampling layer (epipelagic; upper mesopelagic; lower mesopelagic) reached a plateau in OTUs especially at low latitudes (e.g., Tropical and Subtropical) or in the mesopelagic layer. However, spatial diversity patterns, including differences on geographical areas and sampling layers, were reflected in the number of OTUs (Fig 2B).

### Copepod compositions based on presence/absence of OTUs

Water temperature, salinity, and chlorophyll *a* (chl-*a*) concentration varied between the warm waters at low latitudes and cold waters in high latitudes, especially in the epipelagic layer (Fig 1B). These environmental changes led to a significantly different copepod community composition based on the presence/absence of OTUs between warm and cold waters at each sampling layer (*P* < 0.005; Fig 1C; S3 Table). In the cold-water group, the community compositions in the Arctic and Subarctic were clustered into different groups. Copepod community compositions were clearly differentiated by sampling depth between the epipelagic and mesopelagic layers (*P* < 0.005; Fig 1D). The Transition and the California Current regions in the epipelagic layer formed a group distinct from other warm-water groups of the Tropical, North and South subtropical, and Kuroshio regions.

**Fig 3:**
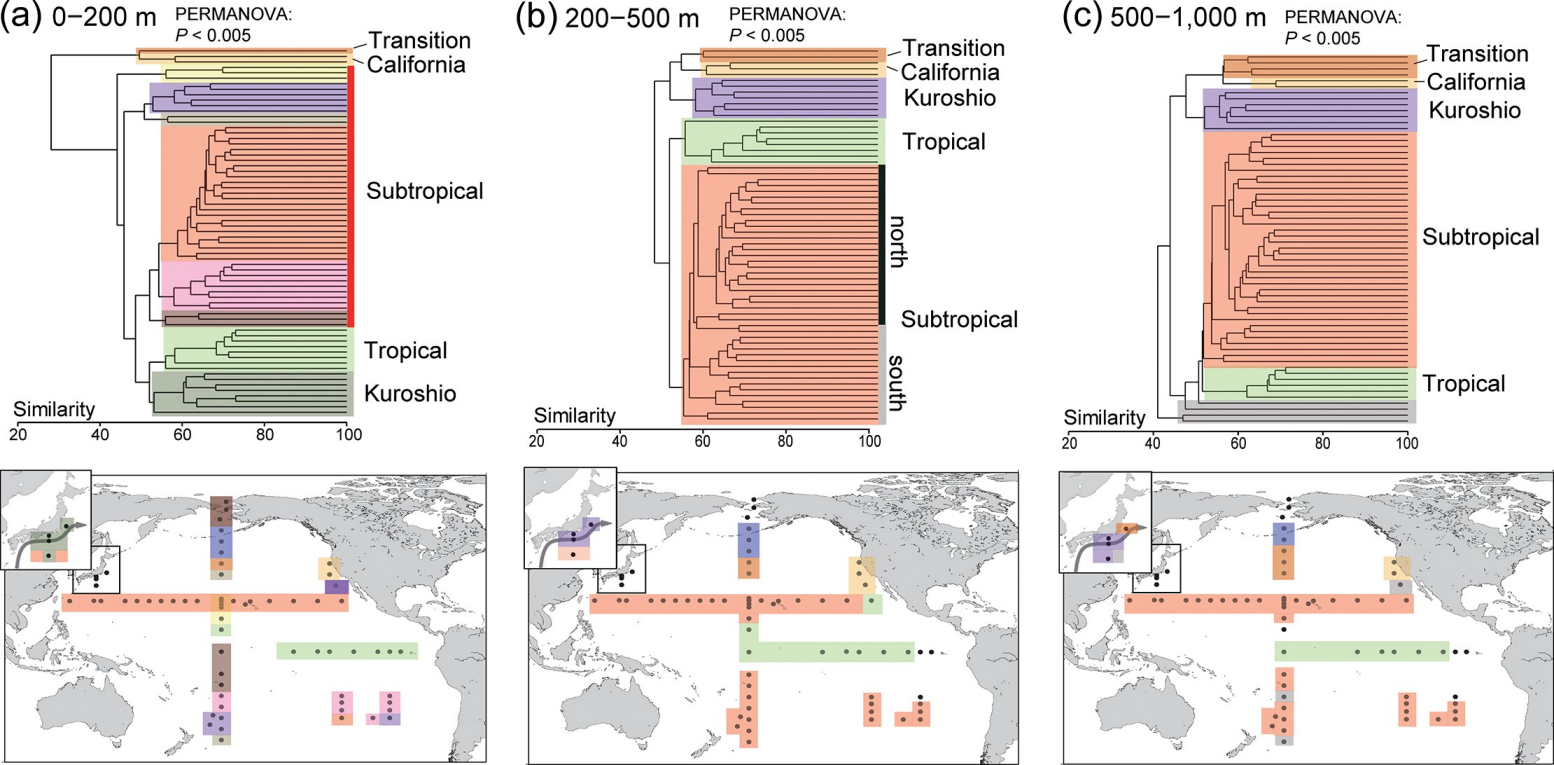
Community compositions at each layer based on presence/absence of operational taxonomic units (OTUs). (a) Epipelagic layer (0–200 m). (b) Upper mesopelagic layer (200–500 m). (c) Lower mesopelagic layer (500–1,000 m). Cluster analyses were performed on warm-water groups. PERMANOVA at each sampling layer was performed on groups color-coded by the region, excluding the unclassified groups (gray) in the lower mesopelagic layer (details in S3 Table). Maps were created by the authors in Generic Mapping Tools version 5 [30].

The clustered groups in the warm-water group clearly corresponded to the respective geographical areas in each sampling layer, namely the Transition, California Current, Kuroshio, subtropical, and tropical regions (Fig 3). The latitudinal subdivisions of community compositions were especially evident in the epipelagic layer even within subtropical regions (Fig 3A; *P* < 0.005), including clear distinction between the North and South Pacific subtropical regions. These community changes were reflected in the best model, which showed that the average water temperature in the epipelagic layer was most strongly related to epipelagic copepod community composition, followed by latitude (Table 1). In addition to latitude, the other geographical factor, longitude, was included in the best model for the epipelagic layer.

**Table 1:** Summary of distance-based linear model (DistLM) analyses based on presence/absence of operational taxonomic units (OTUs). The best model of variables was selected based on the Akaike information criterion (AICc) for all locations in each sampling layer (0–200 m, 200–500 m, and 500–1,000 m) and throughout the sampling layers (0–1,000 m). The pseudo–*F*, *P*–value, and explained variation attributable to the model are indicated for each variable.

	Variable	Pseudo–*F*	*P*–value	Variation (%)
**0–200 m**	Temp. (0–200 m)	14.0	0.001	16.9
AICc: 525.0	Latitude	9.9	0.001	10.5
Variations: 35.5%	DO (0–200 m)	5.7	0.001	5.7
	Longitude	2.5	0.006	2.4
**200–500 m**	Salinity (0–200 m)	7.8	0.001	11.1
AICc: 470.8	Temp. (0–200 m)	7.9	0.001	10.0
Variations: 28.8%	DO (0–200 m)	4.0	0.001	4.8
	Longitude	2.4	0.004	2.9
**500–1,000 m**	Latitude	7.0	0.001	10.3
AICc: 462.0	Temp. (0–200 m)	5.5	0.001	7.6
Variations: 27.5%	DO (0–200 m)	4.1	0.001	5.3
	DO (500–1,000 m)	3.4	0.001	4.3
**0–1,000 m**	Temp. (0–200 m)	16.3	0.001	21.0
AICc: 424.8	Longitude	6.6	0.001	7.9
Variations: 38.9%	DO (0–200 m)	4.9	0.001	5.5
	DO (0–1,000 m)	4.3	0.001	4.5

Temp = average temperature; DO = average dissolved oxygen.

In the upper and lower mesopelagic layers, cluster groups varied by the geographical regions, although variations in community composition were relatively small compared with those in the epipelagic layer (*P* < 0.005; Fig 3B and 3C). The differences between the North and South Pacific subtropical regions were clearer in the upper mesopelagic layer than in the lower mesopelagic layer. The composition in the mesopelagic layer of the Kuroshio region was highly similar to those in the Transition and California Current regions, whereas the structures of the epipelagic layer in Kuroshio was similar to that of the tropical region. The epipelagic environment had a strong effect on the community composition in the mesopelagic layer (Table 1). The best model describing the upper mesopelagic layer included average salinity, water temperature, and dissolved oxygen content of the epipelagic layer as well as longitude. In the lower mesopelagic layer, latitude was the most influential variable, followed by average water temperature, dissolved oxygen content in the epipelagic layer, and the dissolved oxygen content in the lower mesopelagic layer.

**Fig 4:**
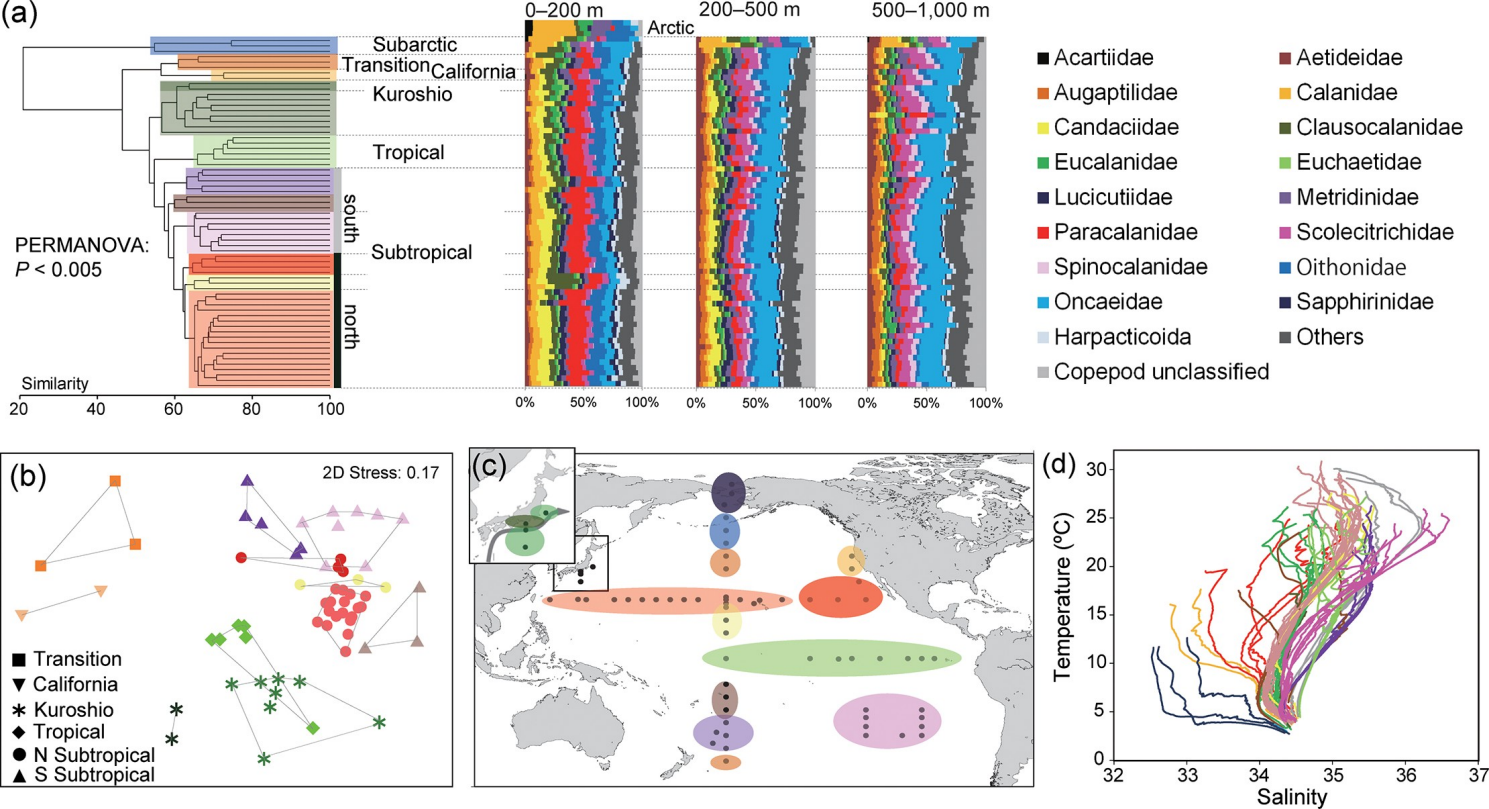
Community compositions based on presence/absence of operational taxonomic units (OTUs) throughout the sampling layers (0–1,000 m). (a) Cluster analysis. PERMANOVA was performed on the cluster groups represented by color (details in S3 Table). Taxonomic compositions of OTUs are shown for each sampling station at each layer. The arctic community is added to the epipelagic layer. (b) Multidimensional scaling analysis for warm-water groups. Cluster groups are connected by lines. (c) Geographic distribution of clustered groups. (d) Comparison of clustered groups and temperature-salinity (T-S) diagram to investigate the effect of water mass structures on copepod community composition. Map was created by the authors in Generic Mapping Tools version 5 [30].

Clear geographic changes were also observed in the cluster groups of community compositions across the sampling layers (0–1,000 m; Fig 4A–4C; *P* < 0.005). In the Kuroshio region, the inshore and oceanic areas of the Kuroshio Current were discriminated. Geographic changes were observed within the subtropical regions and between the North and South Pacific. In addition to latitudinal changes, the western and eastern sides were composed of different clustered groups within North and South Pacific subtropical gyres. The clustered groups corresponded with the distribution of water mass structures, as represented by the profiles of temperature-salinity diagrams (T-S diagrams; Fig 4D). The latitudinal and longitudinal changes through water column were also observed in the best model, with average water temperature in the epipelagic layer as the main factor, followed by longitude (Table 1). There was a clear difference between warm and cold waters in the taxonomic composition of each layer (Fig 4A), but the difference was indistinct with respect to family-level taxonomic composition within warm waters, whereas the proportion of unclassified copepods was high in the mesopelagic layer.

### Copepod community structure based on sequence reads

**Fig 5:**
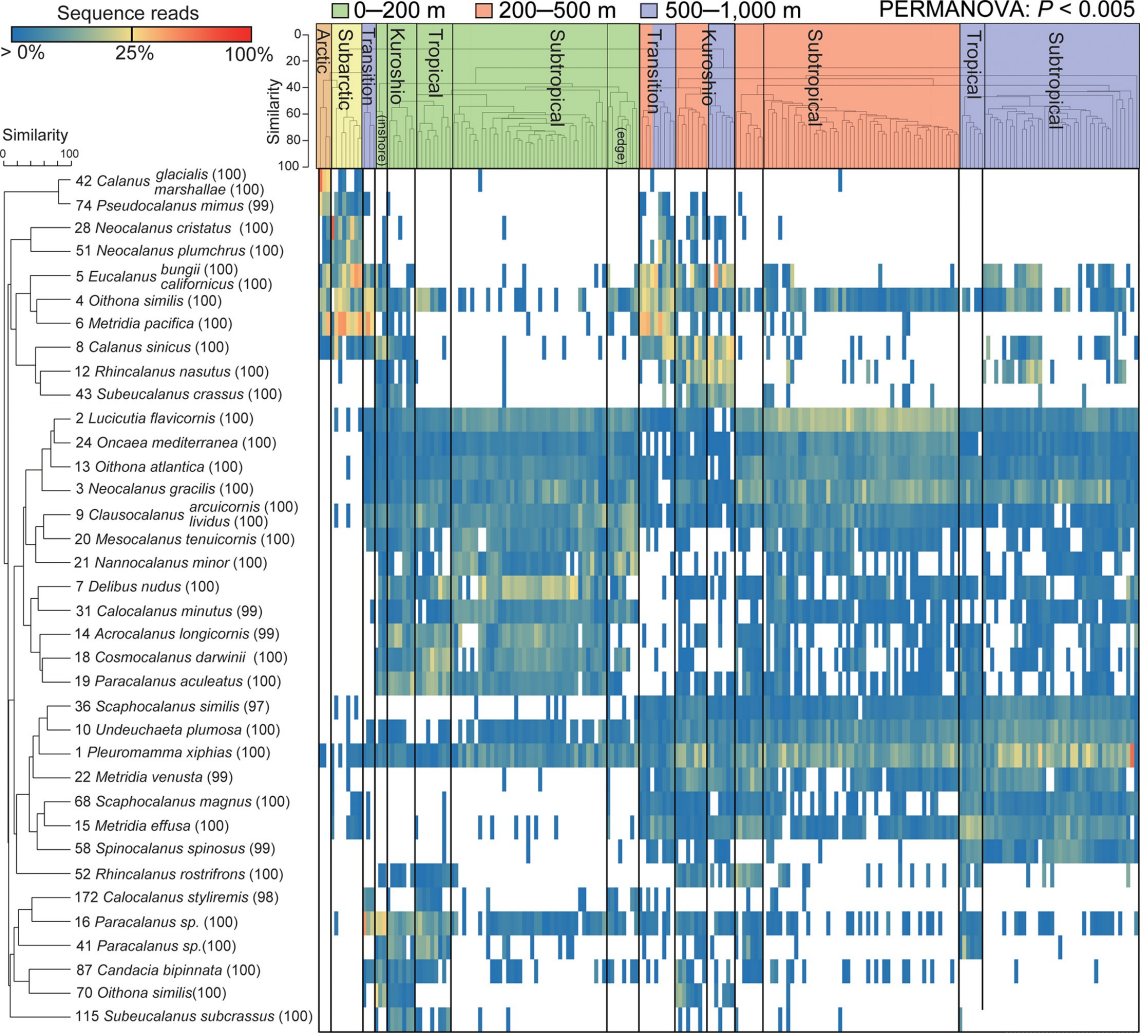
Cluster analysis based on quantitative data and proportions of reads of each major operational taxonomic unit (OTU) within a sample. The cluster analysis was performed on all samples based on square root transformed proportions of sequence reads in OTUs, and the PERMANOVA was performed on clustered groups. The California Current region is included in the Transition region. The OTUs and the best-hit species identified by BLAST search are represented for major OTUs, which were selected by the SIMPER analysis. The numbers in parentheses indicate the similarity percentage of the best-hit species.

The distribution peaks of major OTUs largely affected the large-scale copepod community based on quantitative data using relative proportions of sequence reads. Overall cluster analysis based on sequence reads identified significantly different groups that corresponded with sampling depth and geographical regions (*P* < 0.005; Fig 5). Different epipelagic groups were observed between the inshore and oceanic areas in the Kuroshio region, and between the center and edge of the subtropical gyres. As observed in the analyses based on the OTUs presence/absence, the environmental variables in the epipelagic layer largely affected copepod communities in both the epipelagic and mesopelagic layers (S4 Table). A total of 36 OTUs were selected as major OTUs contributing to the differences among cluster groups (Fig 5). These major OTUs were distinctly different between cold and warm waters, and unique OTUs dominated the arctic and subarctic regions in the cold-water group as well as in the Transition and California Current region. These OTUs were mainly restricted to high latitudes, although some OTUs were present in warm-water mesopelagic layers especially in the Kuroshio region (e.g., OTU 4 with 100% identity to *Oithona similis*). Warm-water OTUs were widely distributed in each layer at low latitudes. The distribution peaks were different in each OTU, even in closely-related taxa, such as in OTU 7 (100% identity to *Delibus nudus*) and OTU 19 (100% identity to *Paracalanus aculeatus*) in the family Paracalanidae in the epipelagic layer and OTU 36 (97% identity to *Scaphocalanus similis*) and OTU 68 (100% identity to *S*. *magnus*) in the family Scolecithrichidae in the mesopelagic layer. Different OTUs characterized the edge (OTUs 9, 20, and 21) and center (OTUs 7, 14, 18, 19, and 31) of subtropical gyres in the epipelagic layer. We also detected specific OTUs with high dominance mainly in the Kuroshio region, including the genera *Subeucalanus* (OTUs 43 and 115) and *Rhincalanus* (OTUs 12 and 52).

### Spatial patterns of the copepod diversity

**Fig 6:**
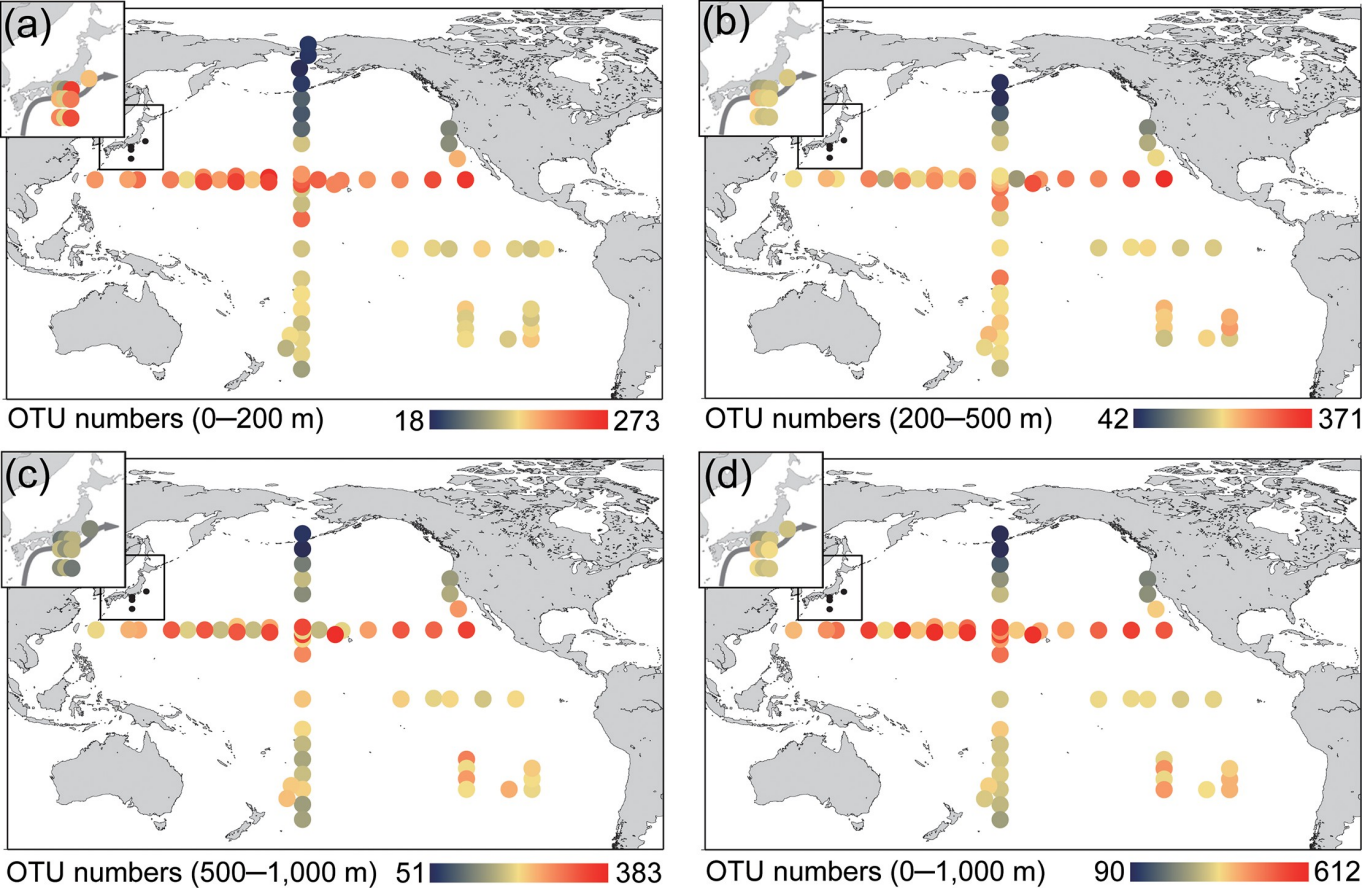
The distribution of copepod diversity at different layers: (a) 0–200 m, (b) 200–500 m, (c) 500–1,000 m, and (d) all sampling layers (0–1,000 m). Colors indicate the number of operational taxonomic units (OTUs). Maps were created by the authors in Generic Mapping Tools version 5 [30].

**Fig 7:**
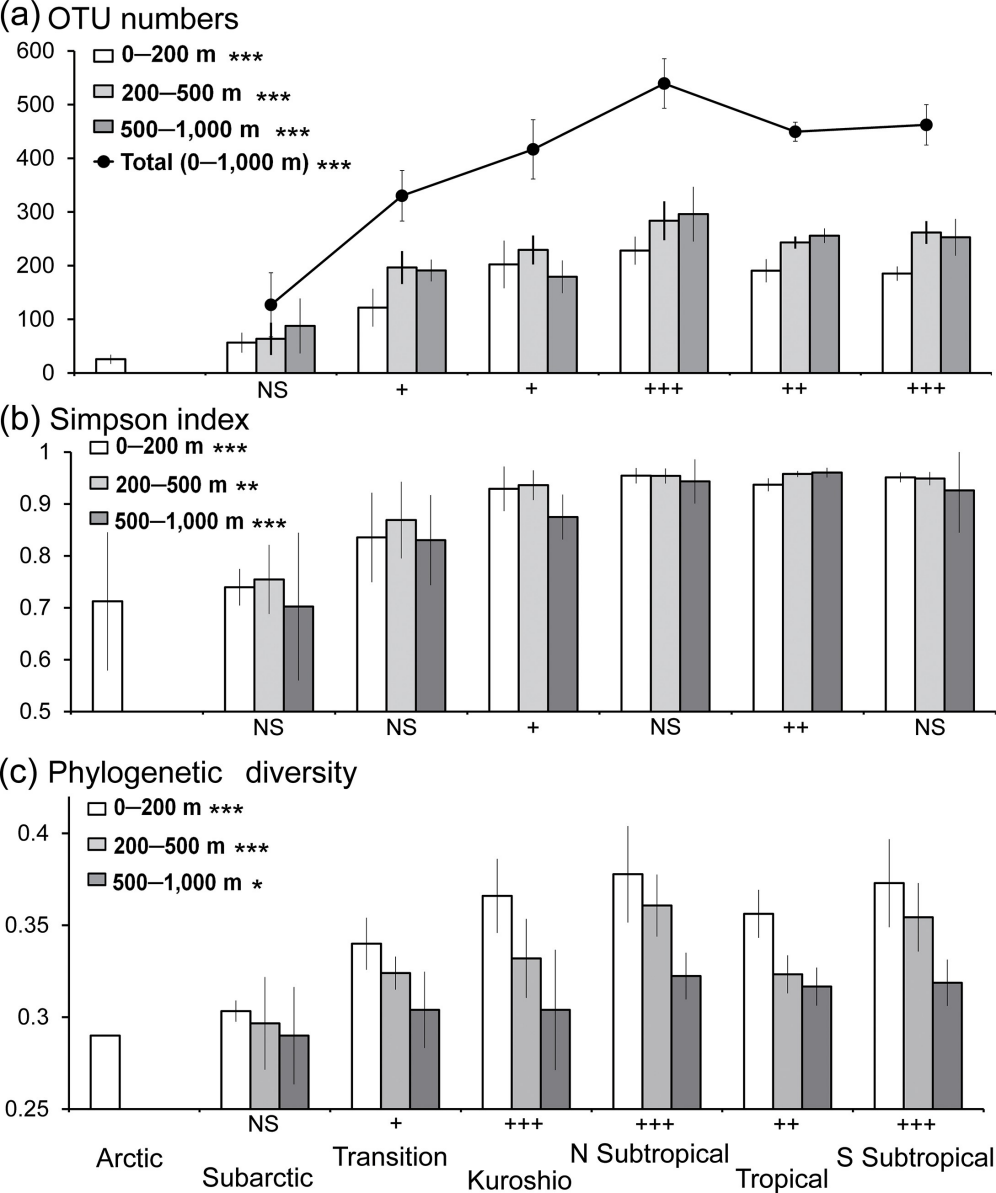
Horizontal and vertical patterns of copepod diversity. (a) Number of operational taxonomic units (OTUs). (b) Simpson diversity index. (c) Phylogenetic diversity. The California Current region is included in Transition region. The diversity parameters were averaged for each region and scale bars indicate standard deviation. NS = not significant; * = *P* < 0.05; ** = *P* < 0.005; *** = *P* < 0.001 based on Kruskal-Wallis tests among geographic regions; + = *P* < 0.05; ++ = *P* < 0.005; +++ = *P* < 0.001 based on Kruskal–Wallis tests among sampling depths. Details of the statistical analyses are listed in S5 Table. Maps were created by the authors in Generic Mapping Tools version 5 [30].

The number of OTUs in each layer showed clear horizontal changes, and OTU number in the low-latitude area of warm water was higher than that in the high-latitude area of cold water (Fig 6). The number of OTUs in the northern and southern hemispheres followed asymmetric latitudinal gradients, and their number in the subtropical North Pacific was significantly higher (*P* < 0.05) than those in other regions (Fig 7A; S5 Table). There was no clear difference between the South subtropical and tropical regions. The OTU number of the Kuroshio epipelagic layer was temporally high, but the diversity of the lower mesopelagic layer was lower than that in tropics and subtropics. These spatial patterns of diversity were observed in the rarefaction curves for each layer (Fig 2). The variable explaining most of the variation in the spatial distributions of OTUs was sea surface temperature (SST) in all layers (Table 2). In the epipelagic layer, SST, followed by salinity, mixed layer depth (MLD), chl-*a*, dissolved oxygen, and latitude were selected the most important parameters in the best model. For the mesopelagic layer, longitude was the second most influential variable after SST, followed by environmental factors of the epipelagic layers, including MLD, dissolved oxygen content, and chl-*a* according to the best model. Unlike the latitudinal changes, the east–west changes in diversity were not clear within the North/South subtropical gyres (S2 Fig), and longitude was not an influential variable according to the best models (Table 2).

**Table 2:** Variables explaining operational taxonomic unit (OTU) numbers. The best model of the variables was selected using a stepwise method in generalized linear model (GLM) analysis, and the pseudo R^2^ and Akaike information criterion (AIC) values are reported for the best models.

	Variable	Estimate	Z value	*P*
**0–200 m**	Intercept	1.53	–0.69	NS
Pseudo R^2^: 0.72	SST	4.28E–02	5.21	< 0.001
AIC: 735.42	Salinity (0–200 m)	1.69E–01	2.54	< 0.05
	MLD	2.38E–03	2.41	< 0.05
	Chl-*a*	–1.28E–01	–2.03	< 0.05
	DO (0–200 m)	–6.11E–02	–1.85	NS
	Latitude	2.40E–03	1.44	NS
**200–500 m**	Intercept	3.35	11.39	< 0.001
Pseudo R^2^: 0.58	SST	4.49E–02	6.74	< 0.001
AIC: 693.35	Longitude	2.49E–03	3.38	< 0.001
	MLD	2.20E–03	2.48	< 0.05
	DO (0–200 m)	6.86E–02	1.92	NS
	Temp (200–500 m)	1.66E–02	1.64	NS
**500–1,000 m**	Intercept	3.48	14.50	< 0.001
Pseudo R^2^: 0.65	SST	4.57E–02	9.56	< 0.001
AIC: 617.34	Longitude	2.80E–03	5.88	< 0.001
	DO (500–1,000 m)	–5.46E–02	–4.05	< 0.001
	DO (0–200 m)	1.11E–01	3.53	< 0.001
**0–1,000 m**	Intercept	10.08	2.76	< 0.01
Pseudo R^2^: 0.77	SST	4.49E–02	9.68	< 0.001
AIC: 626.71	Longitude	2.52E–03	4.75	< 0.001
	DO (0–200 m)	1.51E–01	4.18	< 0.001
	Temp (0–1,000 m)	3.04E–02	3.01	< 0.01
	Chl-*a*	6.39E–01	2.81	< 0.01
	MLD	1.07E–03	2.48	< 0.05
	Salinity (0–1,000 m)	–1.89E–01	–1.74	NS
	DO (0–1,000 m)	–3.63E–02	–1.62	NS

SST = sea surface temperature, Temp = average water temperature (°C), Chl-*a* = average chlorophyll *a* concentration in the epipelagic layer (0–200 m) (μg/L), DO = average dissolved oxygen (ml/L), MLD = mixed layer depth (m), NS = not significant.

The Simpson diversity index (Fig 7B) and phylogenetic diversity (Fig 7C) exhibited a regional patter of high diversity at low latitudes. Unlike the OTUs, the Simpson index values showed an unclear north–south asymmetric pattern for each layer in the tropical and subtropical region. Phylogenetic diversity, based on average genetic distance among OTUs, was high in the North and South subtropical regions for each layer. The regional differences in phylogenetic diversity were especially evident in the epipelagic and upper mesopelagic layers, and a significantly high phylogenetic diversity (*P* < 0.05) was observed in the North subtropical region when compared with other regions (S5 Table).

**Fig 8:**
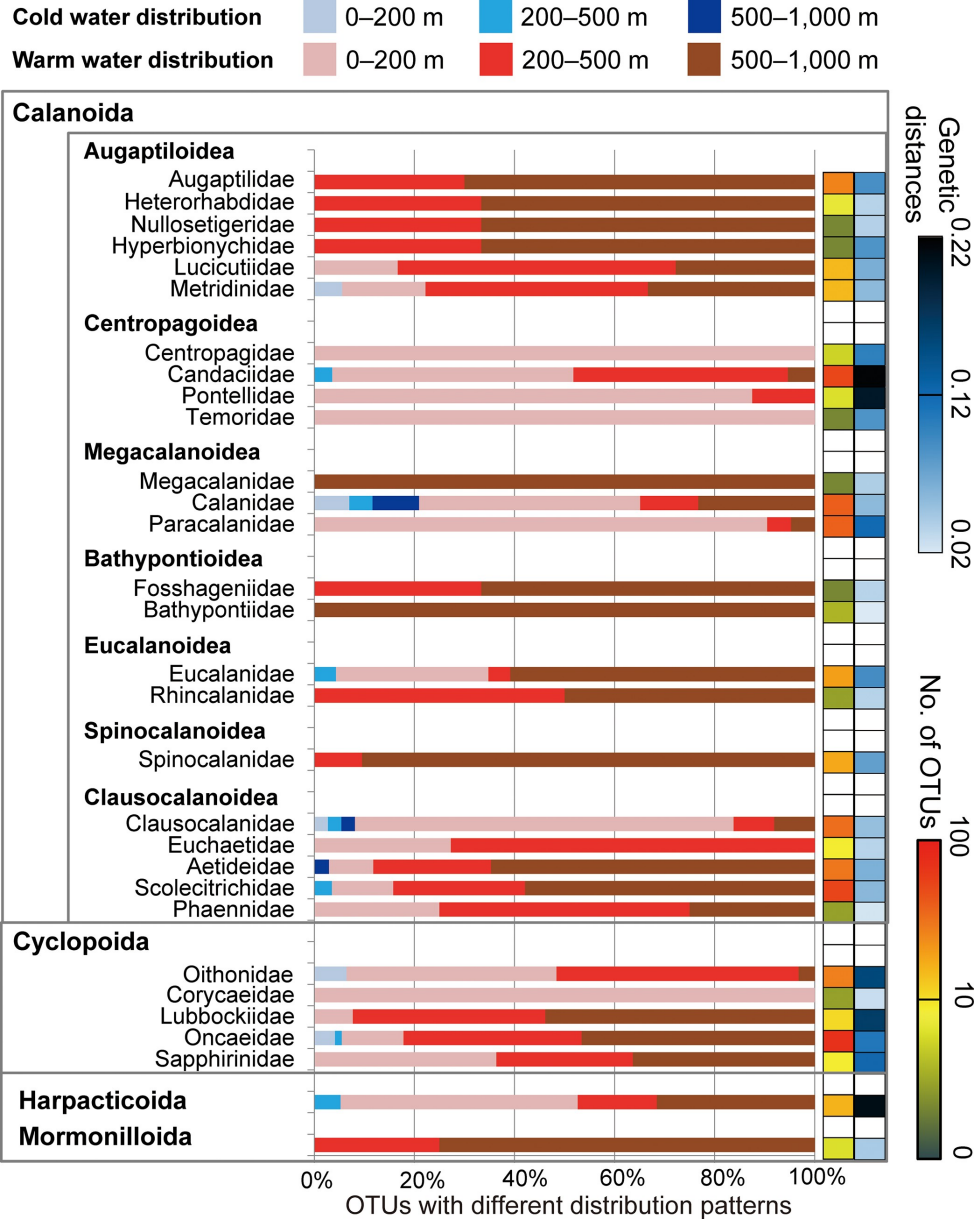
Distribution patterns of copepod operational taxonomic units (OTUs) based on cold- and warm-water groups and water depth (0–200 m, 200–500 m, and 500–1,000 m). Only the OTUs with ≥ 15 sequence reads in a single sample were analyzed, and proportions of numbers of OTUs with different distribution patterns are shown in each taxonomic group. The log scale total OTU numbers and average genetic distance among OTUs are represented as colors for each taxonomic group. The orders Calanoida and Cyclopoida were further classified into families. The superfamily names are indicated for the order Calanoida.

A vertical gradient of diversity was observed, with significantly higher OTU numbers in the mesopelagic than in the epipelagic layer in most geographic regions (Fig 7A; S5 Table). No clear differences in OTU numbers were found between the upper and lower mesopelagic regions in each geographic region. A vertical gradient of diversity was not evident according to the Simpson diversity index (Fig 7B). However, there was a clear vertical gradient in phylogenetic diversity, and it decreased with increasing depth in each geographic region (Fig 7C). The phylogenetic diversity was significantly higher (*P* < 0.05) in the epipelagic layer than in the lower mesopelagic layers in all regions except the Subarctic (S5 Table). Different spatial and vertical distribution patterns of OTUs were associated with taxonomic groups of copepods (Fig 8). High proportions of OTUs in the mesopelagic layer were found in the calanoid superfamilies, such as Augaptiloidea, Bathpontioidea, and Spinocalanoidea, and in the families Megacalanidae, Rhincalanidae, Aetideidae, and Scolecitrichidae, as well as in Lubbockiidae and Oncaeidae in the order Cyclopoida, and in those belonging to the order Mormonilloida. Taxonomic families with high proportions of OTUs distributed in the epipelagic layer included Centropagedae, Pontellidae, Temoridae, Paracalanidae, and Clausocalanidae in the order Calanoida, and Corycaeidae in the order Cyclopoida (Fig 8). The average genetic distances within a family were high in the Centropagoidea and Cyclopoida. Relatively low genetic distances were observed in the Augaptiloidea and Clausocalanoidea.

**Fig 9:**
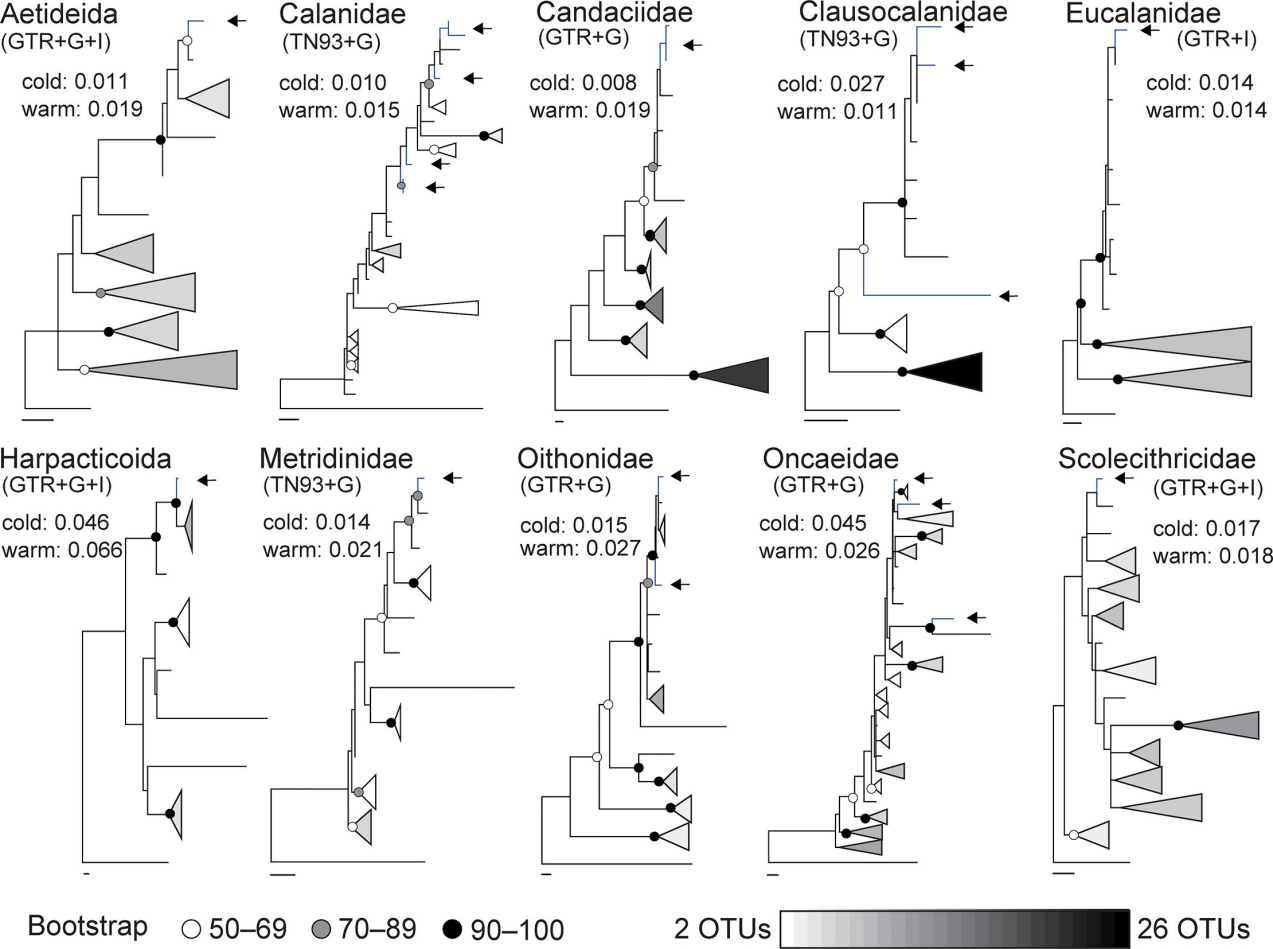
Phylogenetic analysis of taxonomic groups of operational taxonomic units (OTUs) with warm-water and cold-water distributions. OTUs with cold-water distribution are indicated by arrows. Bootstrap values at the nodes are inferred from maximum likelihood analysis (white, grey, black). Genetic lineages containing multiple OTUs with warm-water distribution are presented as triangles for simplicity, and the number of OTUs within each triangle is represented by the white–black spectrum. The length of each triangle corresponds to the length of the deepest node of each lineage. Scale bars indicate 0.02 genetic distances. The average nearest genetic distance is indicated for OTUs with warm-water and cold-water distributions in each taxon. The nucleotide substitution models used are indicated for each taxon (GTR: General Time Reversible; TN93: Tamura-Nei).

The taxa of cold-water OTUs in the arctic and subarctic regions were further analyzed to study the effects of phylogenetic relationships on the distribution patterns of OTUs (Fig 9). In the phylogenetic tree, most of the groups were supported by low bootstrap values; basal positions were mostly occupied by warm-water OTUs with large OTU numbers rather than by cold-water OTUs. The average nearest genetic distances, which could be used as a proxy of divergent time between closely-related OTUs, were lower for cold-water OTUs than for warm-water OTUs, except for Clausocalanidae and Oncaeidae. Similar genetic distance values were observed in cold-water and warm-water OTUs of Eucalanidae.

## Discussion

This study may be first that employed a large number of sequence data to present large-scale distribution patterns of copepods in the epipelagic and mesopelagic layers of the Pacific Ocean. We selected the D2 region of the 28S rRNA because it has higher taxonomic resolution compared with 18S rRNA sequences and higher primer specificity compared with mitochondrial cytochrome oxidase I, the two markers commonly used in zooplankton DNA barcoding [2]. The 28S rRNA gene is also useful in the study of phylogenetic relationships, and it has a high database coverage in various taxonomic groups [31]. We detected a high diversity covering wide range of copepods in the present study. There are possible limitations in a metabarcoding approach, such as overestimation and quantitative biases of OTUs [24]. However, in the present study, the utility of metabarcoding method was validated using mock community analyses, and the rarefaction curve reached a plateau using all sequence data. The present study highlights the hitherto hidden diversity of copepods, because the detected OTUs (1,659) are much larger than the species number detected in the previous studies using morphological classification in the Pacific [20, 32]. However, approximately 2,700 morphological species of marine planktonic copepods are currently known, about 80% of which are calanoids [4]. An additional sampling, including coastal area, other ocean basins and water masses below the mesopelagic layer, would produce more OTUs of copepods.

The major groups, based on both quantitative (sequence reads) and non-quantitative (presence/absence of OTUs) analyses, included distinct communities in the major water masses of arctic, subarctic, transition, subtropical, and tropical regions of the Pacific Ocean. The obtained results were consistent with the results of previous studies based on morphological classifications [12, 20, 32]. The dominance and community composition of copepods are strongly influenced by geological areas (latitude and longitude) with different environmental conditions (e.g., temperature) as indicated by the best model of copepod community compositions. The western boundary of the Kuroshio and California Currents led to similar community structures in the equatorial and transition areas in the epipelagic layer, respectively, because of the ocean current systems in the Pacific. Furthermore, our molecular-based approach revealed latitudinal and east/west subdivisions in copepod compositions, even in environmentally-stable areas within subtropical gyres. Although the North and South Pacific subtropical gyres share a number of common morphological species [18], copepod composition differed between the two gyres. Gene flow of copepods is limited even in oceanic areas lacking evident physical barriers [33], and whole planktonic communities are strongly influenced by distances associated with ocean currents [34]. Because adaptive evolution of zooplankton may occur faster than previously considered [35], the copepod community might have been subdivided through adaptation to local environments with different water masses in the Pacific and Arctic Oceans.

Large-scale patterns in community structure was observed in relatively stable environments of the mesopelagic layer within in the subtropical areas. The changes in copepod composition were especially evident in the epipelagic layer, the zone with large environmental fluctuations. These epipelagic environmental changes also affected the community structure and diversity of copepods in the mesopelagic layer. In addition to the continuous flux of particles from the epipelagic layer, there are mesopelagic copepods with diel vertical migration to the epipelagic layer [36]. Some copepods also migrate to the mesopelagic layer to avoid adverse environmental conditions in the epipelagic layer even in warm waters [37]. A significant change in biomass and trophic efficiency is predicted to occur in mesopelagic nekton and large zooplankton as a result of climate change [38]; thus, interactions between the epipelagic and mesopelagic layers are key regulators of local communities and diversity patterns in mesopelagic copepods.

Pelagic fauna typically displays a latitudinal diversity gradient, with high species numbers in warm low-latitude waters and declining diversity toward higher latitudes [39]. A previous study on prokaryotes and eukaryotes using metabarcoding and image data showed no changes in latitudinal diversity in the mesopelagic layer [29]. In contrast, our metabarcoding approach revealed clear latitudinal diversity gradients in all layers. The diversity index and proportions of sequence reads confirmed the coexistence of many phylogenetically-diverse species in oligotrophic warm waters at low latitudes, and fewer species in the cold, food-rich waters at high latitudes, as reported in the global diversity estimation of epipelagic copepods [15, 40]. Despite the similar environmental conditions in the north and south subtropical gyres, the number of OTUs peaked in the north subtropical region. The North Pacific has a complex water mass structure influenced by a strong western boundary current and the interaction of two major intermediate waters. Therefore, the co-existence of more diversified copepods in complex ecosystems might contribute to higher species richness in the North Pacific than in the South Pacific.

Sequence data combined with OTU distributions provided insights into the mechanisms controlling the large-scale patterns of copepod diversity. Among the several hypotheses that have been proposed to explain latitudinal diversity gradients, our study is mainly consistent with the “out of the tropics” model, in which warm tropical regions are characterized by high speciation and low extinction rates, with most extra-tropical species belonging to lineages that originated in the tropics [41]. Our phylogenetic analyses and nearest genetic distance data support the hypothesis by van der Spoel and Heyman [42] that the central water (subtropical gyre) species are the oldest planktonic taxa and ancestral to cold-water fauna. It has been hypothesized that most of the current zooplankton evolved and assembled after the K-T boundary mass extinction ~65 Ma [43], when the earth’s temperature steadily decreased and latitudinal environmental gradients became stronger [44]. The sea ice in the Arctic is estimated to have appeared about 46 Ma [45], and the abundance of cold-water taxa of planktonic foraminifera increased 36 Ma in the Antarctic [46]. The high-latitude species likely evolved from low-latitude species by adapting to latitudinal environment gradients; hence, the low diversity at high latitude may be contributed to recent emergence of cold-water taxa. The high metabolic rate and short generation time of copepods at high temperatures [1] also might have enabled rapid molecular evolution and high diversity at low latitudes over a long evolutionary history in the historically stable subtropical gyres [47]. Since only short segments of 28S sequences are used in this study, further research using both nuclear and mitochondria markers would improve the resolution and node support of phylogenetic trees and explains the mechanism of latitudinal diversity gradients of pelagic copepods.

The larger numbers of OTUs in the mesopelagic than in the epipelagic layers was consistent with previous studies that documented vertical diversity gradients in zooplankton [48, 49]. Similar to the report by Blanco-Bercial et al. [31], we observed different sequence variations associated with families and superfamilies, which might affect the estimation of vertical gradients of OTUs. These biases were considered to be small, because a relatively high and low sequence variation was detected in copepod families with main distribution in both the epipelagic and mesopelagic layers. The high species richness but low phylogenetic diversity thus indicated co-existence of many genetically similar species in the mesopelagic layer, and these species may have fine-scale segregations of vertical distributions, as reported in some mesopelagic copepods [50, 51]. In the epipelagic layer, on the other hand, the large vertical gradients in environmental factors such as temperature and chl-*a* may be associated with the larger variety of taxonomic groups, because vertical environmental changes are considered an important factor in maintaining diversity of planktonic taxa [52].

Our results suggested that species numbers in specific taxa (e.g., Augaptilidae and Scolecithrichidae) has increased rapidly in recent evolutionary history of copepods, leading to a fine-scale niche partitioning by closely related species in a relatively stable mesopelagic environment. Contrary to our results, the phylogenetic diversity of marine bacteria as lower in shallow water than in deep water [53]. This indicates that the recent rapid increase in species numbers in the mesopelagic layer may be unique in copepods. Although cold-water species are hypothesized to be ancestral to deep water species [42], this evolutionary process was not clearly shown in our metabarcoding study. The evolutionary processes responsible for the differences in mesopelagic and epipelagic waters might be complex and unique to each taxonomic group of oceanic copepods [43]. Copepods may have diversified during their evolution, ultimately leading to the current co-existence of many species at low latitudes, especially in the mesopelagic layer where food sources are limited.

This metabarcoding study of copepods provides a first large-scale overview of both horizontal and vertical changes in copepod community composition and diversity in the Pacific and Arctic Oceans, which have been established through evolutionary processes and maintained in current environmental conditions. The results indicate that both the epipelagic and mesopelagic copepod communities may be vulnerable to anticipated global warming. This highlights the importance of understanding biogeography, community structure, and the mechanisms leading to present biodiversity of copepods in order to monitor the effects of rapid environmental changes. All representative sequences and large-scale distributions of sequence reads for the OTUs produced are available and can be easily included in a future study using the same method. In addition to adding a reference library of DNA barcoding, especially in mesopelagic copepods, a more comprehensive sampling of zooplankton and further development of molecular techniques will enable us to gain a deeper understanding of the driving mechanism of diversity of copepods and other marine pelagic taxa.

## Materials and methods

### Field sampling

Zooplankton samples were collected from 73 sampling stations across the Pacific and Arctic Oceans during 10 research expeditions conducted from 2011 to 2017 (Fig 1A). This study did not involve endangered or protected species, and therefore required no specific permissions for sample collection. Samples in the Pacific Ocean were collected from the epipelagic (0–200 m), upper mesopelagic (200–500 m), and lower mesopelagic (500–1,000 m) layers by vertical tows using a vertical multiple plankton sampler (VMPS) with a 0.5 m^2^ mouth-opening area and 100 μm mesh. The samples in the Kuroshio regions were collected using a VMPS with a 0.25 m^2^ mouth-opening area and 100 μm mesh. For a subset of samples at a depth < 200 m in the Arctic Ocean, we used a North Pacific Standard Plankton (NORPAC) net, with a 100 μm mesh, to collect epipelagic samples by vertical tows from 5 m above the sea floor to the water surface.

A total of 205 environmental samples of bulk zooplankton were collected and immediately preserved in 99% ethanol. The ethanol was replaced within 24 h of initial preservation, and samples were kept at 4°C or -20°C. Detailed information about the samples is listed in S1 Table. Vertical temperature and salinity profiles were obtained using a conductivity, temperature, and depth (CTD) system (SBE-911 plus, Sea-Bird Electronics). Dissolved oxygen content was measured using an SBE-43 dissolved oxygen sensor (Sea-Bird Electronics). Water samples were collected using Niskin bottles attached to the CTD system and then filtered using Whatman GF/F filters for chl-*a* analysis. Chl-*a* was extracted with *N*,*N-*dimethylformamide, and its concentration was analyzed using a Turner fluorometer [54]. MLD at each location was calculated from the depth with a temperature difference of ΔT = 0.2°C from the temperature at 10 m depth [55].

### High-throughput sequencing and bioinformatic analysis

The metabarcoding analysis of copepods was mainly performed according to methods described in a previous paper [28]. Genomic DNA was extracted using a Gentra Puregene Cell and Tissue Kit (QIAGEN) from the 205 environmental zooplankton samples and two mock community samples. The mock communities contained morphologically identified 33 copepod species collected in the Kuroshio region (mock community 1) and 7 copepod species sampled in the North Pacific subtropical gyre (mock community 2) (one individual per species; S1 Fig), which were then destroyed for metabarcoding. The 19 environmental samples at epipelagic layer in the tropical and subtropical Pacific were analyzed [26, 28] using Roche 454 or Illumina MiSeq, and the same DNA or raw sequence data were used in the present study. The DNA concentration of each sample was measured with a Qubit 2.0 Fluorometer (Life Technologies). Library preparations were conducted using three-step PCR and a KOD Plus Version 2 (Toyobo) in a 25 μL reaction volume containing 13 μL distilled water, 2.5 μL 10×buffer, 2.5 μL dNTPs (2 mM), 1.5 μL MgSO_4_ (25 mM), 1.5 μL of each primer (5 μM), 0.5 μL KOD Plus polymerase, and 2 μL template DNA. The first PCR amplified the large ribosomal subunit (LSU) D2 region (approximately 400 bp) using template DNA (1 ng/μL) and a primer pair of LSU Cop-D2F (5ʹ-AGACCGATAGCAAACAAGTAC-3ʹ) and LSU Cop-D2R (5ʹ-GTCCGTGTTTCAAGACGG-3ʹ) [27]. The first PCR cycling protocol consisted of denaturation at 94°C for 2 min, followed by 22 cycles of 10 s denaturation at 98°C, 30 s annealing at 58°C, and 1 min extension at 68°C, with a final extension at 68°C for 7 min. PCR products from the first PCR cycle were diluted (1/20) with distilled water and used as template DNA for the second PCR, and then the process was repeated for the third PCR cycle using the products of the second PCR cycle as template DNA, in which an adaptor and dual-index sequences were attached for sequencing on an Illumina MiSeq. PCR cycles were set at eight cycles with an annealing temperature of 50°C for the second PCR and 59°C for the third PCR. Final PCR products were purified using a QIAquick PCR Purification Kit (QIAGEN), and the concentration of the purified PCR product was measured with a Qubit 2.0 Fluorometer. The quality of the final PCR products was confirmed by an Agilent DNA High Sensitivity Kit on the Bioanalyzer (Agilent). High-throughput sequencing runs was performed on the final PCR products using an Illumina MiSeq, and 2 × 300 bp paired-end sequence reads were obtained.

Bioinformatic analysis was firstly carried out using only data of the mock communities to determine parameters for OTU clustering. Data of the mock communities were also analyzed together with environmental communities to validate the accuracy of the bioinformatic analysis of large sequence dataset. In bioinformatic procedure, raw paired-end reads were initially quality-filtered using Trimmomatic [56] to remove sequences shorter than 100 bp and those with average quality score of <30 in every 30 bp. Paired-end reads were merged in MOTHUR v.1.39.5 [57]. A quality-filtering step in MOTHUR removed sequence reads containing ambiguous bases and no primer sequences. The obtained sequence reads were classified into taxonomic groups using a naïve Bayesian classifier [58] with a threshold > 80% to detect copepod sequences. The reference dataset included manually curated 28S D2 sequences of 257 copepods and 36 other metazoan taxa. Further quality-filtering retained copepod sequences containing ≤6 homopolymers and 300–420 bp after removing primer sequences. An equal number of sequence reads was selected in each sample, and all sequence reads were aligned using the add-fragments option in Multiple Alignment using Fast Fourier Transform (MAFFT) with default settings [59]. Sequences that were not aligned with reference data were removed. We performed a single-linkage pre-clustering, removal of a singleton read, and chimera removal by UCHIME both with and without a reference dataset for the aligned sequences [60]. The final sequence reads were subsampled again, and sequence differences were calculated among sequence reads without considering indels. OTU clustering was performed using OptiClust [61]. We determined similarity threshold and a minimum sequence reads for OTUs following Hirai et al. [28] and the results of the mock community analyses. The representative sequence of each OTU was obtained based on the most abundant sequence reads. The commands and datasets used in bioinformatic analysis are available in Dryad repository. All the following community and diversity analyses were conducted using OTUs, and taxonomy of each OTU was determined at the family level for the orders Calanoida and Cyclopoida (including Poecilostomatoida) and at the order level for other copepod groups.

### Copepod compositions based on presence/absence of OTUs

The broad-scale patterns of copepod compositions were investigated based on the presence or absence of OTUs. Cluster and multidimensional scaling analyses were performed using Bray–Curtis similarity (Sørensen similarity in the case of presence/absence data). Clustered groups at 0–1,000 m depth were compared using T-S diagrams. Permutational analysis of variance (PERMANOVA) was conducted to test the differences among clustered groups, which were determined based on community similarity and geographical regions. PERMANOVAs were conducted using Type III sums of squares and the unrestricted model. The variables explaining OTU composition were analyzed using a distance-based linear model permutation test (DistLM). The best models were selected using a step-wise selection procedure based on Akaike information criterion (AICc). The environmental factors included average water temperature, salinity, dissolved oxygen concentration, chl-*a* concentration, and MLD. The geographical factors were latitude and longitude. In the analysis of epipelagic layer, we used average water temperature from 0 to 200 m, rather than SST, because the former is highly correlated with community composition. In the analyses of mesopelagic layers and throughout the sampling layers (0–1,000 m), environmental valuables at the epipelagic layer were included to evaluate the effects of the upper-layer environments. The community analyses were conducted in PRIMER version 7 with the PERMANOVA+ add-on [62, 63]. All permutation-based tests were conducted using 999 permutations.

### Quantitative analysis using sequence reads of OTUs

In addition to presence/absence of OTUs, quantitative data of sequence reads were analyzed for community structures of copepods and distribution patterns of major OTUs. Cluster analysis and PERMANOVA were conducted for all samples using the square root transformed proportions of sequence reads in OTUs. The major OTUs were selected based on the results of a Similarity Percentage (SIMPER) analysis. The SIMPER analysis can select OTUs contributing to Bray–Curtis similarity within a group or dissimilarity between groups; thus, this analysis is useful for selecting OTUs with distribution peak of sequence reads in a specific cluster group. The top five OTUs or OTUs contributing to at least 70% of community similarity were selected as major OTUs in each cluster group. A BLAST search against the NCBI database was carried out for the representative sequences of major OTUs in order to obtain detailed taxonomic information. Cluster analysis was performed for major OTUs based on their distribution patterns, and spatial distributions of sequence reads were compared among major OTUs.

### Spatial pattern of copepod diversity

The spatial patterns of the OTUs in each sample were compared at each layer and throughout the sampling layers. The evenness for each sample was evaluated using the Simpson diversity index. The phylogenetic diversity was calculated based on the average genetic distance between OTUs, which, in turn, was inferred using the Kimura’s two-parameter nucleotide substitution model, a commonly used genetic distance for zooplankton including copepods [23]. Spatial differences in copepod diversity were evaluated using non-parametric Kruskal–Wallis tests and Dunn’s tests in SPSS 21.0 (IBM Corporation). The effects of environmental variables on OTU numbers were investigated using generalized linear models (GLMs) in R 3.5.0 [64]. We used the same environmental variables as in the DistLM analyses except for water temperature. SST was used as the temperature parameter in the epipelagic layer, because SST is more closely correlated to OTUs than average temperature. Based on the results of the dispersion tests, a negative binomial distribution with the log link function was used for the GLMs for each layer. The best model was selected based on AIC values obtained through backward selection of effective environmental variables. Based on the McFadden’s pseudo R^2^, the goodness of fit for the best model was estimated [65].

### Copepod biogeography and phylogenetic relationships

One of the advantages of metabarcoding analysis is the availability of representative sequences of OTUs. The sequences were used to investigate effects of evolutionary processes on large-scale biogeographic patterns of copepods. An evolutionary process related to latitudinal diversity gradients was particularly focused in the phylogenetic analysis. The biogeographic pattern of each OTU was determined based on distribution of sequence reads. If the highest proportion of sequence reads was observed in the arctic or subarctic region, this OTU was classified as a cold-water OTU. We analyzed OTUs with ≥ 15 sequence reads in a single sample. The proportions of distribution patterns were investigated in each taxonomic group. The taxonomic groups of copepods followed those in Blanco-Bercial et al. [31] and Khodami et al. [66]. The average genetic distances among OTUs were calculated using Kimura’s two-parameter nucleotide substitution model within each taxonomic group. Phylogenetic analyses were conducted in the taxonomic groups containing OTUs with both high-latitude (cold water) and low-latitude distributions (warm water). We added outgroups to each taxonomic group, and sequences were aligned again using MUSCLE [67]. After selection of a best-fit nucleotide substitution model, maximum likelihood analyses were performed with 100 bootstrap replicates to assess nodal support using MEGA 6.0 [68]. The nearest genetic distances of the OTUs were calculated using Kimura’s two-parameter nucleotide substitution model, and average values were compared between OTUs with cold and warm distributions.

## Supporting information

S1 Fig Comparison of operational taxonomic units (OTUs) and reference sequences in mock community analyses. Scale bar indicates genetic distance (p-distance). Note that bioinformatic analysis of mock communities was performed including all environmental communities to validate the accuracy of data analyses.
(TIF)

S2 Fig The distribution pattern of operational taxonomic units (OTUs) at different longitudes. Colors indicate the latitude of each sampling site.
(TIF)

S1 Table Metadata of the environmental zooplankton samples. (PDF)

S2 Table Summary of the preliminary analyses of mock community. These preliminary analyses of mock communities were performed to determine similarity threshold to cluster OTUs and abundance threshold to remove rare and erroneous OTUs. Estimated OTUs are based on reference sequences obtained by Sanger sequencing. An abundance threshold of 8 and a similarity threshold of 98.5%, which are values for environmental community analysis, were used for comparing different values of similarity and abundance threshold, respectively. Target OTUs are identified as copepod species contained in a mock community, and non-target OTUs are other copepod OTUs without high similarity to target species. Note that seven species in mock community 2 were incubated after sampling to remove gut contents, but 33 species without incubation were used for mock community 1. The analyses were based on the preliminary results using only data of the mock communities.
(PDF)

S3 Table Summary of the permutational analysis of variance (PERMANOVA). Copepod community compositions (presence/absence of operational taxonomic units) were compared at each sampling layer between cold-water and warm-water groups and throughout the water column. The effect of sampling layers (epipelagic and mesopelagic) on community composition was investigated for all sampling locations and for warm-water regions. The effect of cluster group on copepod community compositions was investigated for each sampling layer and throughout the water column. The differences among cluster groups based on quantitative data of the sequence reads were also analyzed.
(PDF)

S4 Table Summary of the distance-based linear model permutation test (DistLM) for copepod community based on sequence reads. The best model of environmental variables explaining copepod community composition was selected based on Akaike information criteria (AICc) for all locations in each sampling layer (0–200 m, 200–500 m, and 500–1,000 m). Pseudo–*F*, *P*–value, and explained variation attributable to the model are indicated for each environmental variable.
(PDF)

S5 Table Summary of Kruskal–Wallis and Dunn’s test results for copepod diversity. Operational taxonomic units (OTUs), Simpson index, and phylogenetic diversity were compared among areas at each sampling layer (shallow: 0–200 m, middle: 200–500 m, and deep: 500–1,000 m) and among sampling layers in each area (see Fig 7). If Kruskal–Wallis test was significant, groups with adjusted *P* < 0.05 in pairwise comparisons using Dunn’s tests were listed. Ar = Arctic, Sa = Subarctic, Tra = Transition, Ns = North subtropical gyre, Tro = Tropical, Ss = South subtropical.
(PDF)
