# Supplementary figures and images for: Large-scale metabarcoding analysis of epipelagic and mesopelagic copepods in the Pacific
Source: PLoS One. 2020 May 14;15(5):e0233189. doi: 10.1371/journal.pone.0233189 (PMC7224477; doi:10.1371/journal.pone.0233189)

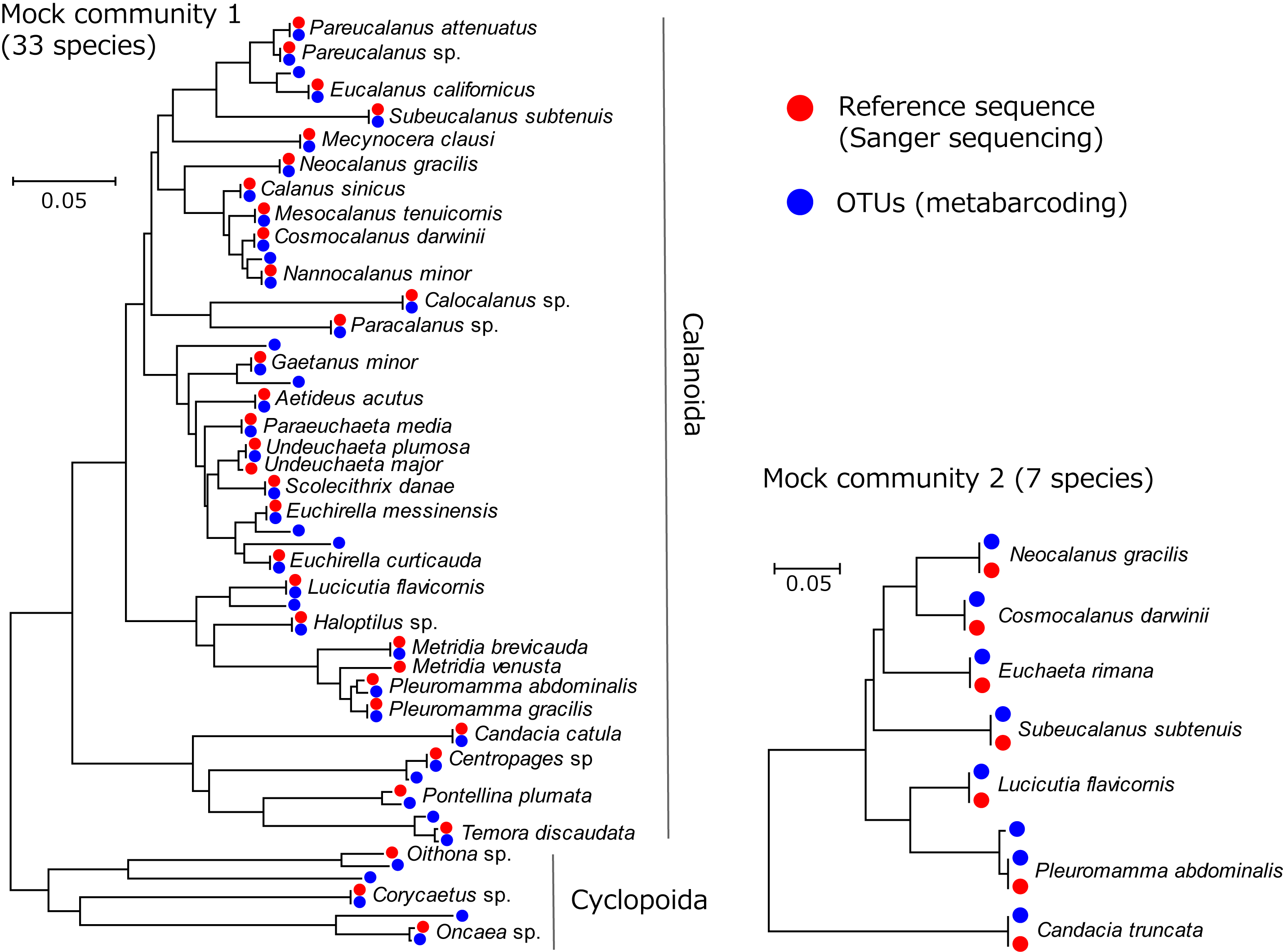

Supplement: S1 Fig — Scale bar indicates genetic distance (p-distance). Note that bioinformatic analysis of mock communities was performed including all environmental communities to validate the accuracy of data analyses. (TIF) [file pone.0233189.s001.tif]

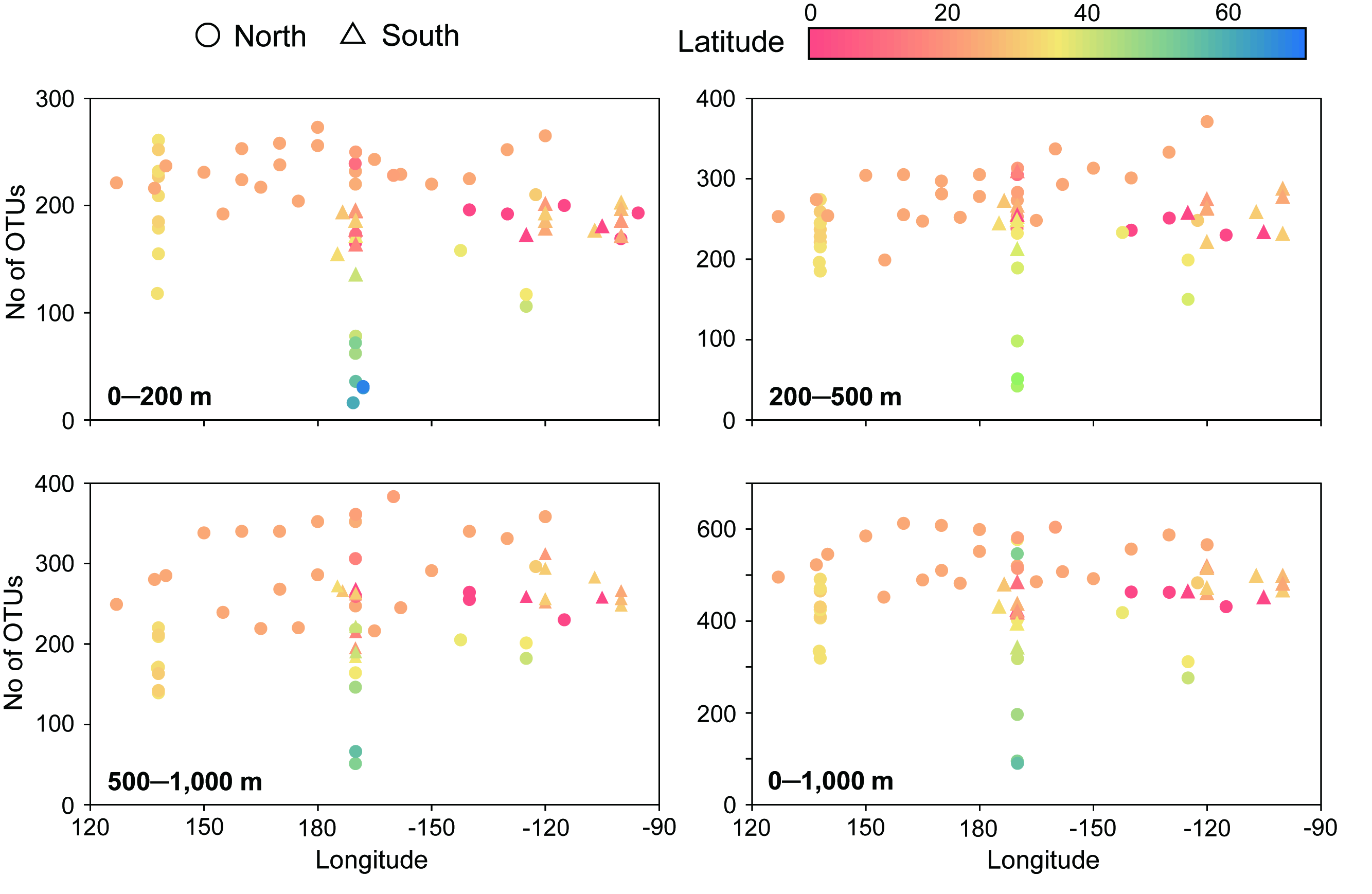

Supplement: S2 Fig — Colors indicate the latitude of each sampling site. (TIF) [file pone.0233189.s002.tif]
