# Supplementary material for: Large-scale metabarcoding analysis of epipelagic and mesopelagic copepods in the Pacific
Source: PLoS One. 2020 May 14;15(5):e0233189. doi: 10.1371/journal.pone.0233189 (PMC7224477; doi:10.1371/journal.pone.0233189)
Supplement: S1 Table — (PDF) [file pone.0233189.s003.pdf]

S1 Table. Metadata of the environmental zooplankton samples.

	Cruise	Station	Latitude	Longitude	Date	Time (local time)	Sampling gear	Depth	OTUs	Biosample accession
1	SY-11-05	S01	33° 52.0´N	137° 44.0´E	5-May-11	14:29–15:07	VMPS 3000D	0–200 m	118	SAMD00140688
2	(Soyo–maru)							200–500 m	196	SAMD00140689
3								500–1,000 m	170	SAMD00140690
4		K01	32° 54.6´N	138° 00.5´E	17-May-11	10:26–10:43	VMPS 3000D	0–200 m	179	SAMD00140691
5						12:40–13:26		200–500 m	237	SAMD00140692
6								500–1,000 m	171	SAMD00140693
7		C3100	31° 00.0´N	137° 59.9´E	9-May-11	9:25–9:56	VMPS 3000D	0–200 m	185	SAMD00140694
8								200–500 m	221	SAMD00140695
9								500–1,000 m	209	SAMD00140696
10	KH-11-10	st1	22° 58.7´N	155° 00.5´E	Dec. 5, 2011	17:19–17:43	VMPS 6000D	0–200 m	192	SAMD00140697
11	(Hakuho–maru)							200–500 m	199	SAMD00140698
12								500–1,000 m	239	SAMD00140699
13		st2	23° 00.5´N	159° 59.0´E	Dec. 6, 2011	23:06–23:31	VMPS 6000D	0–200 m	224	SAMD00140700
14								200–500 m	255	SAMD00140701
15		st3	23° 00.5´N	165° 00.0´E	Dec. 9, 2011	19:00–19:26	VMPS 6000D	0–200 m	217	SAMD00140702
16								200–500 m	247	SAMD00140703
17								500–1,000 m	219	SAMD00140704
18		st4A	23° 00.1´N	170° 00.1´E	Dec. 10, 2011	22:17–22:43	VMPS 6000D	0–200 m	238	SAMD00140705
19								200–500 m	281	SAMD00140706
20								500–1,000 m	268	SAMD00140707
21		st4B	23° 00.2´N	174° 54.8´E	Dec. 12, 2011	04:42–05:07	VMPS 6000D	0–200 m	204	SAMD00140708
22								200–500 m	252	SAMD00140709
23								500–1,000 m	220	SAMD00140710
24		St5	23° 00.8´N	179° 59.9´E	Dec. 14, 2011	03:02–03:28	VMPS 6000D	0–200 m	273	SAMD00140711
25								200–500 m	278	SAMD00140712

26							500–1,000 m	286	SAMD00140713
27	st6	22° 59.2´N	170° 01.2´W	Dec. 15, 2011	17:55–18:20	VMPS 6000D	0–200 m	232	SAMD00140714
28							200–500 m	273	SAMD00140715
29							500–1,000 m	247	SAMD00140716
30	st7	23° 00.6´N	165° 01.2´W	Dec. 16, 2011	23:25–23:57	VMPS 6000D	0–200 m	243	SAMD00140717
31							200–500 m	248	SAMD00140718
32							500–1,000 m	216	SAMD00140719
33	St8	22° 46.8´N	158° 05.7´W	Dec. 19, 2011	01:54–02:20	VMPS 6000D	0–200 m	229	SAMD00140720
34							200–500 m	293	SAMD00140721
35							500–1,000 m	245	SAMD00140722
36	st14	19° 59.9'S	120° 01.3'W	Jan. 6, 2012	14:11–14:36	VMPS 6000D	0–200 m	202	SAMD00140723
37							200–500 m	275	SAMD00140724
38							500–1,000 m	312	SAMD00140725
39	st15	23° 00.1´S	120° 00.3´W	Jan. 7, 2012	20:53–21:19	VMPS 6000D	0–200 m	179	SAMD00140727
40							200–500 m	263	SAMD00140728
41							500–1,000 m	252	SAMD00140729
42	st16	26° 28.7´S	120° 00.2´W	Jan. 10, 2012	01:37–02:02	VMPS 6000D	0–200 m	186	SAMD00140730
43							200–500 m	264	SAMD00140731
44							500–1,000 m	294	SAMD00140732
45	st17	30° 00.0´S	119° 59.0´W	Jan. 12, 2012	02:56–03:20	VMPS 6000D	0–200 m	193	SAMD00140733
46							200–500 m	222	SAMD00140734
47							500–1,000 m	256	SAMD00140735
48	st18	29° 59.9'S	107° 00.2'W	Jan. 13, 2012	15:49–16:13	VMPS 6000D	0–200 m	177	SAMD00140736
49							200–500 m	259	SAMD00140737
50							500–1,000 m	283	SAMD00140738
51	st19	29° 59.9´S	100° 00.2´W	Jan. 15, 2012	19:37–20:01	VMPS 6000D	0–200 m	203	SAMD00140739
52							200–500 m	232	SAMD00140740

53								500–1,000 m	248	SAMD00140741
54		st20	26° 30.0´S	100° 00.0´W	Jan. 16, 2012	19:17–19:42	VMPS 6000D	0–200 m	197	SAMD00140742
55								200–500 m	288	SAMD00140743
56								500–1,000 m	256	SAMD00140744
57		st21	22° 59.8'S	100° 00.7'W	Jan. 18, 2012	16:02–16:27	VMPS 6000D	0–200 m	172	SAMD00140745
58								200–500 m	278	SAMD00140746
59								500–1,000 m	266	SAMD00140747
60		st22	20° 00.0'S	100° 00.0'W	Jan. 20, 2012	15:11–15:16	VMPS 6000D	0–200 m	186	SAMD00140748
61	KH-12-01	st1	0° 00.2´N	95° 30.0´W	Feb. 2, 2012	04:07–04:13	VMPS 6000D	0–200 m	193	SAMD00140749
62	(Hakuho–maru)	st2	0° 00.4'N	100° 04.5'W	Feb. 3, 2002	09:28–09:34	VMPS 6000D	0–200 m	169	SAMD00140750
63		st3	0° 00.0'S	105° 00.1'W	Feb. 4, 2012	15:17–15:41	VMPS 6000D	0–200 m	181	SAMD00140751
64								200–500 m	234	SAMD00140752
65								500–1,000 m	258	SAMD00140753
66		st5	0° 00.1´N	115° 00.0´W	Feb. 7, 2012	01:13–01:39	VMPS 6000D	0–200 m	200	SAMD00140754
67								200–500 m	230	SAMD00140755
68								500–1,000 m	230	SAMD00140756
69		st7	0° 00.1'S	125° 00.1'W	Feb. 9, 2012	10:32–10:57	VMPS 6000D	0–200 m	173	SAMD00140757
70								200–500 m	258	SAMD00140758
71								500–1,000 m	259	SAMD00140759
72		st8	0° 02.2'N	129° 59.3'W	Feb. 11, 2012	13:03–13:28	VMPS 6000D	0–200 m	192	SAMD00140760
73								200–500 m	251	SAMD00140761
74								500–1,000 m	255	SAMD00140762
75		st10 (night)	0° 00.2´N	139° 59.8´W	Feb. 13, 2012	21:10–21:35	VMPS 6000D	0–200 m	196	SAMD00140763
76								200–500 m	236	SAMD00140764
77								500–1,000 m	264	SAMD00140765
78	KS-13-T2	TK1(night)	35° 50.0´N	142° 20´E	Oct. 14, 2013	no data	VMPS 6000D	0–200 m	158	SAMD00140766
79	(Shinsei–maru)							200–500 m	233	SAMD00140767

80								500–1,000 m	205	SAMD00140768
81	KH-13-7	st1	0° 01.2´N	170° 00.6´W	Dec. 23, 2013	4:37–4:47?	VMPS 6000D	0–200 m	172	SAMD00140769
82	(Hakuho–maru)							200–500 m	255	SAMD00140770
83								500–1,000 m	269	SAMD00140771
84		st3	10° 00.6´S	170° 00.0´W	Dec. 27, 2013	21:13–21:35	VMPS 6000D	0–200 m	178	SAMD00140772
85								200–500 m	310	SAMD00140773
86								500–1,000 m	262	SAMD00140774
87		st4	14° 59.7´S	170° 00.3´W	Jan. 2, 2014	4:16–4:38	VMPS 6000D	0–200 m	195	SAMD00140775
88								200–500 m	253	SAMD00140776
89								500–1,000 m	215	SAMD00140777
90		st5	19° 57.1´S	170° 00.5´W	Jan. 5, 2014	0:43–1:03	VMPS 6000D	0–200 m	196	SAMD00140778
91								200–500 m	256	SAMD00140779
92								500–1,000 m	195	SAMD00140780
93		st6	24° 59.7´S	169° 59.9´W	Jan. 7, 2014	0:49–1:10	VMPS 6000D	0–200 m	164	SAMD00140781
94								200–500 m	267	SAMD00140782
95								500–1,000 m	222	SAMD00140783
96		st7	30° 01.0´S	170° 00.2´W	Jan. 9, 2014	20:35–20:54	VMPS 6000D	0–200 m	186	SAMD00140784
97								200–500 m	255	SAMD00140785
98								500–1,000 m	263	SAMD00140786
99		st8	34° 59.5´S	170° 02.7´W	Jan. 11-12, 2014	23:53–0:12	VMPS 6000D	0–200 m	186	SAMD00140787
100								200–500 m	249	SAMD00140788
101								500–1,000 m	184	SAMD00140789
102		st9	40° 00.4´S	169° 59.4´W	Jan. 13, 2014	6:24–6:43	VMPS 6000D	0–200 m	136	SAMD00140790
103								200–500 m	213	SAMD00140791
104								500–1,000 m	190	SAMD00140792
105		st Ⅰ	28° 45.5'S	173° 26.3'W	Jan. 17, 2014	20:09–20:30	VMPS 6000D	0–200 m	194	SAMD00140793

106								200–500 m	273	SAMD00140794
107								500–1,000 m	266	SAMD00140795
108		stU	33° 03.9'S	174° 47.1'W	Jan. 16, 2014	16:33–16:53	VMPS 6000D	0–200 m	155	SAMD00140796
109								200–500 m	245	SAMD00140797
110								500–1,000 m	272	SAMD00140798
111	KH-14-3	st3	10° 02.0´N	170° 08.0´W	Jul. 6, 2014	21:13–21:35	VMPS 6000D	0–200 m	239	SAMD00140799
112	(Hakuho–maru)							200–500 m	238	SAMD00140800
113		st4	15° 00.8´N	170° 01.2´W	Jul. 8, 2014	1:36–1:57	VMPS 6000D	0–200 m	165	SAMD00140801
114								200–500 m	305	SAMD00140802
115								500–1,000 m	306	SAMD00140803
116		st5	20° 00.6´N	169° 58.4´W	Jul. 9, 2014	3:29–3:39	VMPS 6000D	0–200 m	173	SAMD00140804
117								200–500 m	313	SAMD00140805
118								500–1,000 m	259	SAMD00140806
119		st6	24° 00.0´N	170° 00.3´W	Jul. 10, 2014	14:32–14:54	VMPS 6000D	0–200 m	220	SAMD00140807
120								200–500 m	253	SAMD00140808
121								500–1,000 m	352	SAMD00140809
122		st8	35° 01.9´N	170° 01.5´W	Jul. 22, 2014	0:16–0:25	VMPS 6000D	0–200 m	172	SAMD00140810
123								200–500 m	232	SAMD00140811
124								500–1,000 m	164	SAMD00140812
125		st9	40° 00.7´N	169° 59.7´W	Jul. 23, 2014	5:58–6:19	VMPS 6000D	0–200 m	78	SAMD00140813
126								200–500 m	189	SAMD00140814
127								500–1,000 m	218	SAMD00140815
128		st10	45° 00.4´N	170° 00.9´W	Jul. 24, 2014	9:38–10:00	VMPS 6000D	0–200 m	62	SAMD00140816
129								200–500 m	98	SAMD00140817
130								500–1,000 m	146	SAMD00140818
131		st11	50° 00.1´N	170° 02.0´W	Jul. 26, 2014	1:15–1:35	VMPS 6000D	0–200 m	72	SAMD00140819
132								200–500 m	42	SAMD00140820

133								500–1,000 m	51	SAMD00140821
134		st12	54° 59.6´N	169° 59.9´W	Jul. 27, 2014	15:45–16:05	VMPS 6000D	0–200 m	36	SAMD00140822
135								200–500 m	51	SAMD00140823
136								500–1,000 m	66	SAMD00140824
137		st13	59° 59.4´N	170° 39.5´W	Jul. 28, 2014	16:16–16:19	Norpac twin	0–62 m	16	SAMD00140825
138		st14	64° 15.2´N	167° 59.5´W	Jul. 29, 2014	13:35–13:37	Norpac twin	0–32 m	30	SAMD00140826
139		st15	68° 00.1´N	168° 00.1´W	Jul. 30, 2014	8:22–8:25	Norpac twin	0–50 m	31	SAMD00140827
140	SY-15-01	C3000	29° 59.6'N	137° 59.2'E	Jan. 22, 2015	0:53–1:15	VMPS 3000D	0–200 m	227	SAMD00140828
141	(Soyo–maru)							200–500 m	259	SAMD00140829
142								500–1,000 m	163	SAMD00140830
143		C3300	33° 00.0'N	138° 00.0'E	Jan. 26, 2015	3:28–2:57	VMPS 3000D	0–200 m	209	SAMD00140831
144								200–500 m	274	SAMD00140832
145								500–1,000 m	220	SAMD00140833
146		C3345	33° 45.0'N	138° 00.0'E	Jan. 27, 2015	18:16–18:40	VMPS 3000D	0–200 m	155	SAMD00140834
147								200–500 m	185	SAMD00140835
148								500–1,000 m	139	SAMD00140836
149	SY-15-10	C3400	34° 00.1'N	137 59.6'E	Oct. 24, 2015	18:21–18:49	VMPS 3000D	0–200 m	261	SAMD00140837
150	(Soyo–maru)							200–500 m	215	SAMD00140838
151								500–1,000 m	210	SAMD00140839
152		C3315	33° 15.0'N	138° 00.3'E	Oct. 26, 2015	20:01–20:40	VMPS 3000D	0–200 m	232	SAMD00140840
153								200–500 m	245	SAMD00140841
154								500–1,000 m	211	SAMD00140842
155		C3030	30° 30.0'N	138° 00.1'E	Oct. 28, 2015	21:07–21:42	VMPS 3000D	0–200 m	252	SAMD00140843
156								200–500 m	228	SAMD00140844
157								500–1,000 m	142	SAMD00140845
158	KH-16-7	N04	23° 01.4'N	126° 59.2'E	Dec. 13, 2016	11:56–12:19	VMPS 6000D	0–200 m	221	SAMD00140846
159	(Hakuho–maru)							200–500 m	253	SAMD00140847

160								500–1,000 m	249	SAMD00140848
161	KH-17-4	st2	39° 59.4'N	124° 59.8'W	Aug. 14, 2017	21:41–22:05	VMPS 6000D	0–200 m	106	SAMD00140849
162	(Hakuho–maru)							200–500 m	150	SAMD00140850
163								500–1,000 m	182	SAMD00140851
164		st3	35° 00.0'N	125° 00.6'W	Aug. 16, 2017	21:56–22:21	VMPS 6000D	0–200 m	117	SAMD00140852
165								200–500 m	199	SAMD00140853
166								500–1,000 m	201	SAMD00140854
167		st4	29° 59.6'N	122° 29.6'W	Aug. 18, 2017	20:47–21:11	VMPS 6000D	0–200 m	210	SAMD00140855
168								200–500 m	248	SAMD00140856
169								500–1,000 m	296	SAMD00140857
170		st6	23° 00.3'N	120° 00.9'W	Aug. 20, 2017	21:13–21:37	VMPS 6000D	0–200 m	265	SAMD00140858
171								200–500 m	371	SAMD00140859
172								500–1,000 m	358	SAMD00140860
173		st7	23° 00.6'N	130° 00.4'W	Aug. 25, 2017	19:50–20:19	VMPS 6000D	0–200 m	252	SAMD00140861
174								200–500 m	333	SAMD00140862
175								500–1,000 m	331	SAMD00140863
176		st8	23° 00.2'N	140° 00.6'W	Aug. 28, 2017	20:42–21:06	VMPS 6000D	0–200 m	225	SAMD00140864
177								200–500 m	301	SAMD00140865
178								500–1,000 m	340	SAMD00140866
179		st9	23° 00.2'N	149° 59.5'W	Aug. 31, 2017	20:15–20:39	VMPS 6000D	0–200 m	220	SAMD00140867
180								200–500 m	313	SAMD00140868
181								500–1,000 m	291	SAMD00140869
182		st10	21° 30.2'N	160° 00.6'W	Sep. 2, 2017	21:09–21:33	VMPS 6000D	0–200 m	228	SAMD00140870
183								200–500 m	337	SAMD00140871
184								500–1,000 m	383	SAMD00140872
185		st11	21° 31.6'N	169° 59.5'W	Sep. 12, 2017	21:15–21:41	VMPS 6000D	0–200 m	250	SAMD00140873
186								200–500 m	283	SAMD00140874

187							500–1,000 m	361	SAMD00140875
188	st12	22° 59.7'N	180° 00.0'E	Sep. 16, 2017	20:53–21:18	VMPS 6000D	0–200 m	256	SAMD00140876
189							200–500 m	305	SAMD00140877
190							500–1,000 m	352	SAMD00140878
191	st13	23° 01.0'N	169° 57.7'E	Sep. 20, 2017	20:55–21:17	VMPS 6000D	0–200 m	258	SAMD00140879
192							200–500 m	297	SAMD00140880
193							500–1,000 m	340	SAMD00140881
194	st14	22° 59.8'N	159° 59.7'E	Sep. 23, 2017	19:10–19:34	VMPS 6000D	0–200 m	253	SAMD00140882
195							200–500 m	305	SAMD00140883
196							500–1,000 m	340	SAMD00140884
197	st15	23° 00.9'N	150° 02.4'E	Sep. 27, 2017	22:59–23:24	VMPS 6000D	0–200 m	231	SAMD00140885
198							200–500 m	304	SAMD00140886
199							500–1,000 m	338	SAMD00140887
200	st16	22° 59.8'N	139° 59.5'E	Sep. 29, 2017	22:14–22:38	VMPS 6000D	0–200 m	237	SAMD00140888
201							200–500 m	254	SAMD00140889
202							500–1,000 m	285	SAMD00140890
203	st17	23° 08.1'N	137° 00.4'E	Oct. 1, 2017	21:57–22:22	VMPS 6000D	0–200 m	216	SAMD00140891
204							200–500 m	274	SAMD00140892
205							500–1,000 m	280	SAMD00140893
