# Supplementary material for: Large-scale metabarcoding analysis of epipelagic and mesopelagic copepods in the Pacific
Source: PLoS One. 2020 May 14;15(5):e0233189. doi: 10.1371/journal.pone.0233189 (PMC7224477; doi:10.1371/journal.pone.0233189)
Supplement: S2 Table — These preliminary analyses of mock communities were performed to determine similarity threshold to cluster OTUs and abundance threshold to remove rare and erroneous OTUs. Estimated OTUs are based on reference sequences obtained by Sanger sequencing. An abundance threshold of 8 and a similarity threshold of 98.5%, which are values for environmental community analysis, were used for comparing different values of similarity and abundance threshold, respectively. Target OTUs are identified as copepod species contained in a mock community, and non-target OTUs are other copepod OTUs without high similarity to target species. Note that seven species in mock community 2 were incubated after sampling to remove gut contents, but 33 species without incubation were used for mock community 1. The analyses were based on the preliminary results using only data of the mock communities. (PDF) [file pone.0233189.s004.pdf]

S2 Table. Summary of the preliminary analyses of mock community. These preliminary analyses of mock communities were performed to determine similarity threshold to cluster OTUs and abundance threshold to remove rare and erroneous OTUs. Estimated numbers of OTUs are based on reference sequences obtained by Sanger sequencing. An abundance threshold of 8 and a similarity threshold of 98.5%, which are values for environmental community analysis, were used for comparing different values of similarity and abundance threshold, respectively. Target OTUs are numbers of OTUs identified as copepod species contained in a mock community, and non-target OTUs are other copepod OTUs without high similarity to target species. Note that seven species in mock community 2 were incubated after sampling to remove gut contents, but 33 species without incubation were used for mock community 1. The analyses were based on the preliminary results only using data of mock communities.

	Similarity (abundance threshold 8)				Abundance threshold (similarity 98.5%)			
	99%	98.5%	98%	97%	2	3	8	15
**Mock 1 (33 morphological species)**								
Estimated no. of OTUs (Sanger)	32	30	29	26	30	30	30	30
Target OTUs	42	34	29	26	48	42	34	32
Undetected species	2	2	2	2	1	1	2	2
Non-target OTUs	11	9	10	7	36	12	9	8
Total OTUs	53	43	39	33	84	54	43	40
**Mock 2 (7 morphological species)**								
Estimated no. of OTUs (Sanger)	7	7	7	7	7	7	7	7
Target OTUs	12	8	7	7	11	11	8	8
Undetected species	0	0	0	0	0	0	0	0
Non-target OTUs	0	0	0	0	8	3	0	0
Total OTUs	12	8	7	7	19	14	8	8
