# Supplementary material for: Large-scale metabarcoding analysis of epipelagic and mesopelagic copepods in the Pacific
Source: PLoS One. 2020 May 14;15(5):e0233189. doi: 10.1371/journal.pone.0233189 (PMC7224477; doi:10.1371/journal.pone.0233189)
Supplement: S3 Table — Copepod community compositions (presence/absence of operational taxonomic units) were compared at each sampling layer between cold-water and warm-water groups and throughout the water column. The effect of sampling layers (epipelagic and mesopelagic) on community composition was investigated for all sampling locations and for warm-water regions. The effect of cluster group on copepod community compositions was investigated for each sampling layer and throughout the water column. The differences among cluster groups based on quantitative data of the sequence reads were also analyzed. (PDF) [file pone.0233189.s005.pdf]

S3 Table. Summary of the permutational analysis of variance (PERMANOVA). Copepod community compositions (presence/absence of Operational Taxonomic Units) were compared at each sampling layer between cold-water and warm-water groups and throughout the water column. The effect of sampling layers (epipelagic and mesopelagic) on community composition was investigated for all sampling locations and for warm-water regions. The effect of cluster group on copepod community compositions was investigated for each sampling layer and throughout the water column. The differences among cluster groups based on quantitative data of the sequence reads were also analyzed.

		Df	Pseudo-*F*	*P*-value
Warm/cold waters	0–200 m	1	10.8	0.001
	200–500 m	1	7.2	0.001
	500–1,000 m	1	6.0	0.002
	0-1,000 m	1	14.3	0.001
Epipelagic/mesopelagic	All	1	27.1	0.001
	Warm	1	32.2	0.001
Cluster group (presence/absence)	0–200 m	11	6.3	0.001
	200–500 m	5	4.4	0.001
	500–1,000 m	5	4.5	0.001
	0–1,000 m	11	6.8	0.001
Cluster group (sequence reads)	All samples	13	24	0.001
