# Supplementary material for: Large-scale metabarcoding analysis of epipelagic and mesopelagic copepods in the Pacific
Source: PLoS One. 2020 May 14;15(5):e0233189. doi: 10.1371/journal.pone.0233189 (PMC7224477; doi:10.1371/journal.pone.0233189)
Supplement: S4 Table — The best model of environmental variables explaining copepod community composition was selected based on Akaike information criteria (AICc) for all locations in each sampling layer (0–200 m, 200–500 m, and 500–1,000 m). Pseudo–F, P–value, and explained variation attributable to the model are indicated for each environmental variable. (PDF) [file pone.0233189.s006.pdf]

S4 Table. Summary of the distance-based linear model permutation test (DistLM) for copepod community based on sequence reads. The best model of environmental variables explaining copepod community composition was selected based on Akaike information criteria (AICc) for all locations in each sampling layer (0–200 m, 200–500 m, and 500–1,000 m). Pseudo–*F*, *P*–value, and explained variation attributable to the model are indicated for each environmental variable.

	Variable	Pseudo–*F*	*P*–value	Variation (%)
**0–200 m**	Temp. (0–200 m)	15.0	0.001	17.8
AICc: 531.1	Latitude	10.1	0.001	10.6
Variations: 40.1%	DO (0–200 m)	7.5	0.001	7.1
	Salinity (0–200 m)	2.5	0.013	2.3
	Longitude	2.4	0.023	2.1
**200–500 m**	Temp. (0–200 m)	9.3	0.001	12.9
AICc: 473.5	Salinity (0–200 m)	8.2	0.001	10.2
Variations: 32.4%	DO (0–200 m)	5.3	0.001	6.2
	Longitude	2.8	0.001	3.1
**500–1,000 m**	Latitude	7.5	0.001	11
AICc: 475.6	Temp. (0–200 m)	5.2	0.001	7
Variations: 34.5%	DO (0–200 m)	4.2	0.001	5.4
	DO (500–1,000 m)	4.0	0.001	5
	MLD	2.6	0.003	3.1
	Salinity (0–200 m)	2.5	0.002	2.9

Temp = average temperature; DO = average dissolved oxygen.
