# Supplementary material for: Large-scale metabarcoding analysis of epipelagic and mesopelagic copepods in the Pacific
Source: PLoS One. 2020 May 14;15(5):e0233189. doi: 10.1371/journal.pone.0233189 (PMC7224477; doi:10.1371/journal.pone.0233189)
Supplement: S5 Table — Operational taxonomic units (OTUs), Simpson index, and phylogenetic diversity were compared among areas at each sampling layer (shallow: 0–200 m, middle: 200–500 m, and deep: 500–1,000 m) and among sampling layers in each area (see Fig 7). If Kruskal–Wallis test was significant, groups with adjusted P < 0.05 in pairwise comparisons using Dunn’s tests were listed. Ar = Arctic, Sa = Subarctic, Tra = Transition, Ns = North subtropical gyre, Tro = Tropical, Ss = South subtropical. (PDF) [file pone.0233189.s007.pdf]

S5 Table. Summary of Kruskal–Wallis and Dunn's test results for copepod diversity. Operational taxonomic units (OTUs), Simpson index, and phylogenetic diversity were compared among areas at each sampling layer (shallow: 0–200 m, middle: 200–500 m, and deep: 500–1,000 m) and among sampling layers in each area (see Fig. 7). If Kruskal–Wallis test was significant, groups with adjusted $P < 0.05$ in pairwise comparisons using Dunn's tests were listed. Ar = Arctic, Sa = Subarctic, Tra = Transition, Ns = North subtropical gyre, Tro = Tropical, Ss = South subtropical.

	Groups	Kruskal–Wallis	Groups with adjusted $P < 0.05$ in Dann's test
OTU numbers (region)	0–200 m	$P < 0.001$	Ns–Ar, Ns–Sa, Ns–Tro, Ns–Ss
	200–500 m	$P < 0.001$	Ns–Sa, Ns–Tra, Ns–Tro, Ns–Ss
	500–1,000 m	$P < 0.001$	Ns–Sa, Ns–Tra, Ns–Ku, Ss–Ku
	Total OTUs	$P < 0.001$	Ns–Sa, Ns–Tra, Ns–Ku, Ns–Tro, Ns–Ss
Simpson (region)	0–200 m	$P < 0.001$	Ns–Ar, Ns–Sa, Ns–Tra, Ss–Tra
	200–500 m	$P < 0.005$	Sa–Ns, Sa–Tro
	500–1,000 m	$P < 0.001$	Sa–Ns, Sa–Tro, Tra–Ns, Tra–Tro, Ku–Ns, Ku–Tro
Phylogenetic diversity (region)	0–200 m	$P < 0.001$	Ar–Ns, Sa–Ns
	200–500 m	$P < 0.001$	Ns–Sa, Ns–Tra, Ns–Tro, Ns–Ku
	500–1,000 m	$P < 0.05$	
OTU numbers (depth)	Subarctic	Not significant	
	Transition	$P < 0.05$	Shallow–Middle
	Kuroshio	$P < 0.05$	Middle–Deep
	N subtropical	$P < 0.001$	Shallow–Middle, Shallow–Deep
	Tropical	$P < 0.005$	Shallow–Deep
	S subtropical	$P < 0.001$	Shallow–Middle, Shallow–Deep

Simpson (depth)	Subarctic	Not significant	
	Transition	Not significant	
	Kuroshio	$P < 0.05$	Middle–Deep
	N subtropical	Not significant	
	Tropical	$P < 0.005$	Shallow–Middle, Shallow–Deep
	S subtropical	Not significant	
Phylogenetic diversity (depth)	Subarctic	Not significant	
	Transition	$P < 0.05$	Shallow–Deep
	Kuroshio	$P < 0.001$	Shallow–Deep
	N subtropical	$P < 0.001$	Shallow–Middle, Shallow–Deep
	Tropical	$P < 0.005$	Shallow–Deep
	S subtropical	$P < 0.001$	Shallow–Middle, Shallow–Deep
